# Multi-targeted azacoumarin–cyanocinnamate hybrids induce G_2_/M arrest and apoptosis *via* tubulin, and COX-2/VEGFR modulation: insights from *in vitro* mechanistic basis and *in vivo* validation

**DOI:** 10.1039/d5md00484e

**Published:** 2025-08-22

**Authors:** Manar A. El-Zend, Ibrahim M. El-Deen, Rawda M. Mansour, Tarek A. Yousef, Amal Abdullah Alrashidi, Essa M. Saied

**Affiliations:** a Department of Chemistry, Faculty of Science, Port Said University Port Said Egypt m.elzend@sci.psu.edu.eg ieldeen@yahoo.com rawdamohamed226@gmail.com; b College of Science, Chemistry Department, Imam Mohammad Ibn Saud Islamic University (IMSIU) Riyadh 11623 Saudi Arabia tayousef@imamu.edu.sa; c Department of Pharmaceutical Sciences, College of Pharmacy, Princess Nourah bint Abdulrahman University P.O. Box 84428 Riyadh 11671 Saudi Arabia aaalrashidi@pnu.edu.sa; d Chemistry Department, Faculty of Science, Suez Canal University Ismailia 41522 Egypt essa.saied@science.suez.edu.eg; e Institute for Chemistry, Humboldt Universität zu Berlin 12489 Berlin Germany

## Abstract

Cancer remains a significant global health concern, with breast cancer ranking among the leading causes of cancer-related mortality in women. In pursuit of multi-targeted anticancer agents, we designed and synthesized a novel series of 7-hydroxy azacoumarin–α-cyanocinnamate hybrids and evaluated their therapeutic potential through comprehensive *in vitro* and *in vivo* studies. Structural characterization was confirmed using NMR, IR, and elemental analysis. Among the synthesized compounds, compound 7 exhibited the most potent cytotoxic activity against MCF-7 cells (IC_50_ = 7.65 μM) and MDA-MB-231 (IC_50_ = 9.7 ± 1.15 μM), with notable selectivity over non-tumorigenic MCF-10A cells (IC_50_ = 52.02 μM), as compared to the reference drug doxorubicin. Mechanistic *in vitro* investigations revealed that compound 7 induced G_2_/M phase arrest and apoptosis, accompanied by upregulation of pro-apoptotic markers (Bax, p53) and suppression of Bcl-2. Additionally, compound 7 significantly inhibited tubulin polymerization and demonstrated marked antioxidant activity in the FRAP assay (IC_50_ = 144.71 μM), as well as selective COX-2 inhibition (IC_50_ = 1.264 μM, SI = 5.93). *In vivo* evaluation using the Ehrlich ascites carcinoma (EAC) model confirmed its anticancer efficacy, with 85.92% reduction in viable EAC cells and substantial tumor volume suppression at 10 mg kg^−1^. Notably, compound 7 mitigated EAC-induced hepatorenal toxicity by restoring liver and kidney biomarkers and reducing oxidative stress and lipid peroxidation. Furthermore, it significantly downregulated pro-inflammatory (TNF-α) and angiogenic (VEGFR-II) markers while preserving normal tissue histoarchitecture. Collectively, these findings highlight compound 7 as a promising multi-functional lead candidate with cytotoxic, antioxidant, anti-inflammatory, and anti-angiogenic activities, meriting further development in cancer therapeutics.

## Abstract 2

Cancer remains a significant global health concern, with breast cancer ranking among the leading causes of cancer-related mortality in women.

## Graphical abstract

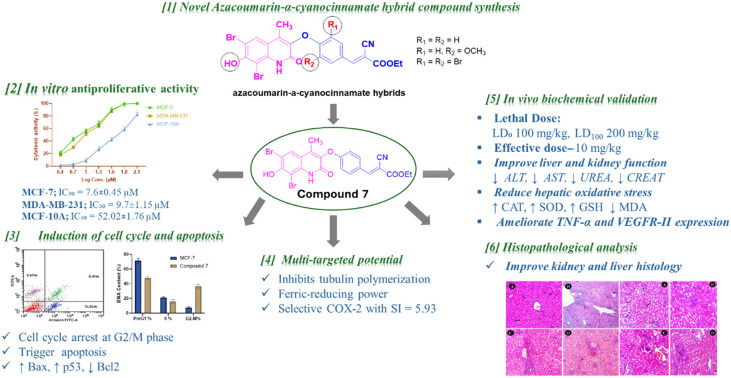

## 1. Introduction

Cancer remains a major global health burden and a leading cause of death, with breast cancer ranking as the most commonly diagnosed malignancy in women and a principal cause of cancer-related mortality.^1^ Regardless of significant developments in therapeutic strategies, including conventional chemotherapies, the management of cancer continues to face challenges such as multidrug resistance, off-target toxicity, and lack of specificity. These challenges have driven the development of multi-targeted therapies aimed at disrupting multiple pathways involved in tumor progression, survival, and metastasis.^2,3^ Several interrelated biological processes are disturbed in cancer, including uncontrolled cell proliferation, enhanced oxidative stress, activation of angiogenesis, attenuation of apoptosis, and unregulated cell cycle.^4^ In this regard, several anticancer drugs, *e.g.*, paclitaxel and vincristine, act mainly by targeting tubulin, leading to mitotic arrest and apoptotic cell death.^5^ Alongside this, the modulation of pro- and anti-apoptotic signals remains a key target for effective cancer therapy, as dysregulation of apoptotic gene expression (*e.g.*, Bcl2, p53, Bax genes) significantly impacts tumor survival.^6^ Similarly, the regulation of oxidative stress is considered as a potential strategy for attenuating the uncontrolled progression within the tumor. Therefore, the development of anticancer agents with antioxidant features may improve their efficacy by modulating the redox balance either by triggering antioxidant defense, such as glutathione (GSH), superoxide dismutase (SOD), and catalase (CAT), or selectively augmenting oxidative damage in tumor cells.^7^ In parallel, inflammation is closely relevant to oxidative stress in cancer. Several studies reported the overexpression of cyclooxygenases in several cancers, which has been associated with angiogenesis, promoting prostaglandin-mediated survival, and impaired immune responses.^8,9^ Similarly, angiogenesis, particularly the VEGF/VEGFR axis, plays a critical role in promoting neovascularization to support tumor growth. Anti-angiogenic therapies targeting VEGFR pathways, such as bevacizumab, have demonstrated significant clinical benefit.^10^ Collectively, these facts further support the demand for a novel class of multitarget agents that can efficiently and synergistically modulate apoptotic, oxidative, angiogenesis, and inflammatory pathways.

**Fig. 1:**
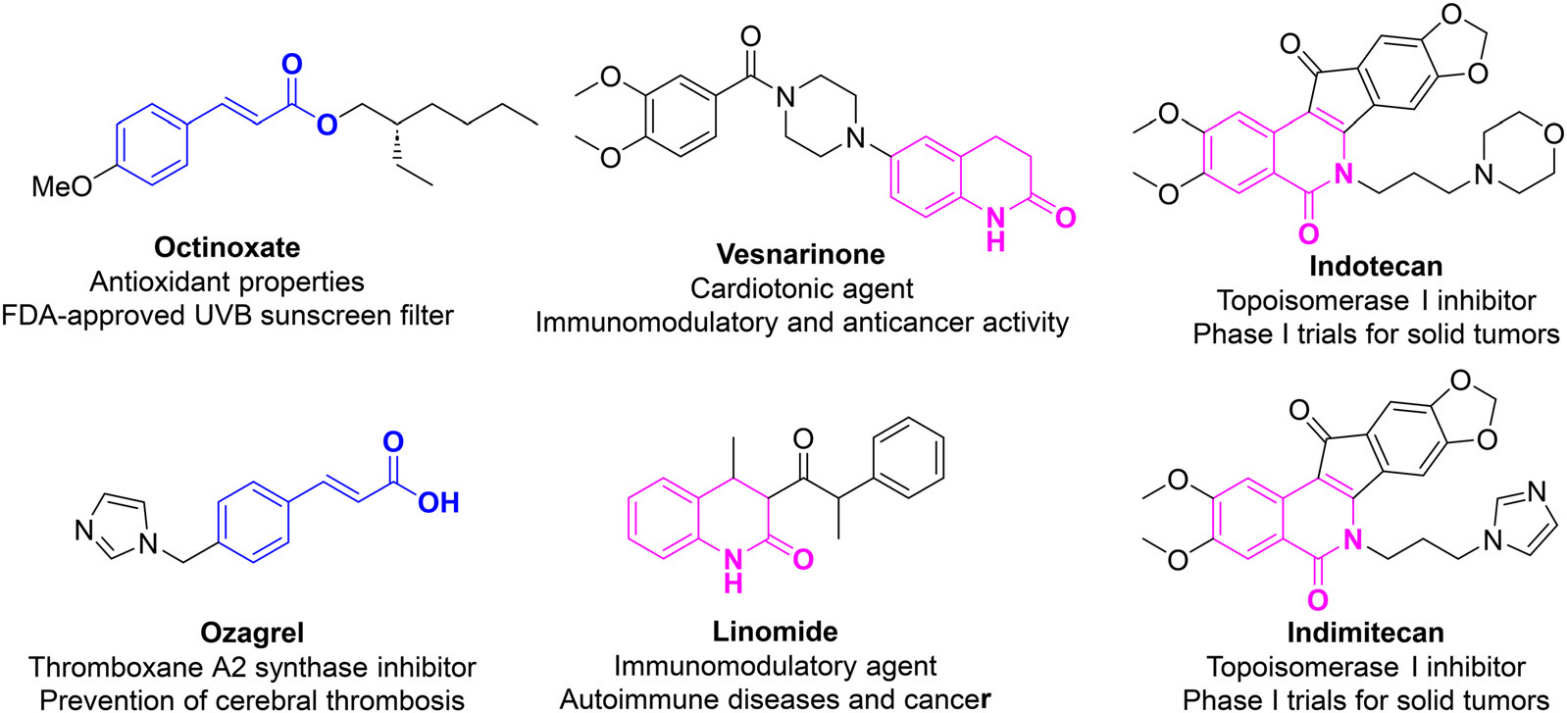
Representative chemical structures of clinically approved drugs with cinnamic acid- and 2-quinolinone-based structures.

Recently, the quest for innovative multi-target anticancer agents has been directed to molecular hybrid-based compounds, which combine two or more bioactive pharmacophores with complementary mechanisms of action into a single molecular scaffold. The molecular hybridization strategy has demonstrated the success of providing novel bioactive compounds with enhanced efficacy, reduced toxicity, and multi-targeted biological profiles.^11–16^ Among the privileged scaffolds, coumarins, especially azacoumarins (2-quinolones), and cinnamic acid derivatives have emerged as privileged scaffolds in anticancer drug discovery. Azacoumarins are a class of heterocyclic compounds known for their broad pharmacological spectrum, including antioxidants, antineoplastic, anticancer, and antimicrobial.^17–19^ Their structure has been incorporated into several clinically approved anticancer drugs, such as irinotecan, topotecan, linomide, and vesnarinone (Fig. 1).^20–22^ The anticancer effects of this class have been linked to their ability to interfere with several key oncogenic mechanisms, including cell cycle arrest, mitochondria-mediated apoptosis, and angiogenesis.^23–28^ Recently, several azacoumarin-based hybrid compounds have demonstrated activity against a range of tumor cell lines, making them attractive candidates for further structural optimization.^17,18,24^ On the other hand, cinnamic acid derivatives are naturally occurring phenylpropanoids, commonly found in fruits, vegetables, and medicinal plants. Their α,β-unsaturated carbonyl system acts as a Michael acceptor to covalently interact with nucleophilic residues in targeted proteins, making them possess a wide range of bioactivities.^29,30^ The cinnamate-based compounds have demonstrated the ability to attenuate several pathways involved in tumor progression, such as histone deacetylases (HDACs), NF-κB, EGFR, and matrix metalloproteinases (MMPs). Thus, the cinnamate scaffold has been recognized in drug development, such as octinoxate and ozagrel (Fig. 1).^31^ Moreover, cinnamates are known to exert free radical scavenging effects, modulate cellular redox status, and interfere with inflammatory cascades.^29,30,32^

**Fig. 2:**
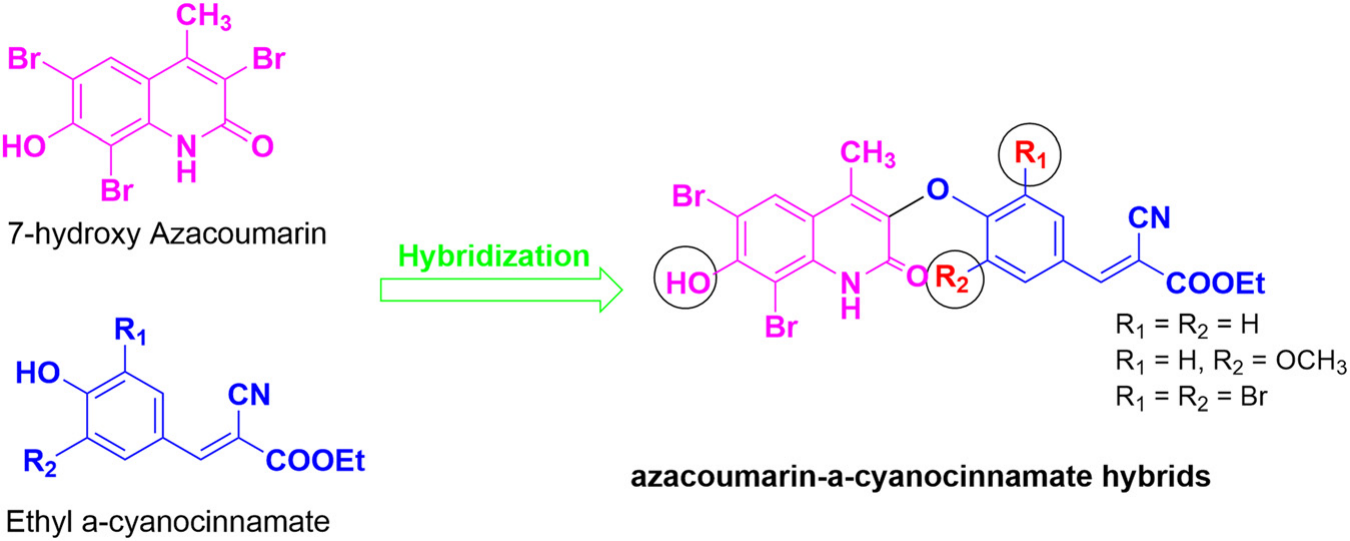
Schematic representation for the strategy adopted to design the envisioned 7-hydroxy-azacoumarin–α-cyanocinnamate hybrids.

Given the therapeutic potential of azacoumarin and cinnamate scaffolds and our ongoing research to discover novel anticancer agents,^33–38^ we report in this study the synthesis and evaluation of a novel set of 7-hydroxy azacoumarin–α-cyanocinnamate hybrids as multi-targeted anticancer agents (Fig. 2). The antiproliferative activity and selectivity of envisioned compounds were examined against MCF-7, MDA-MB 231, and MCF-10A cells. Through extensive *in vitro* and *in vivo* biological assessments, we characterized the potential of this scaffold as a multi-targeted anticancer agent capable of modulating various biological processes in cancer. Toward this, we conducted a comprehensive mechanistic investigation of its effects on tubulin polymerization, apoptosis regulation, cell cycle progression, redox balance, inflammatory signaling, and angiogenesis, alongside *in vivo* validation in the Ehrlich ascites carcinoma (EAC) model.

## 2. Results and discussion

### 2.1 Chemistry

**Scheme 1:**
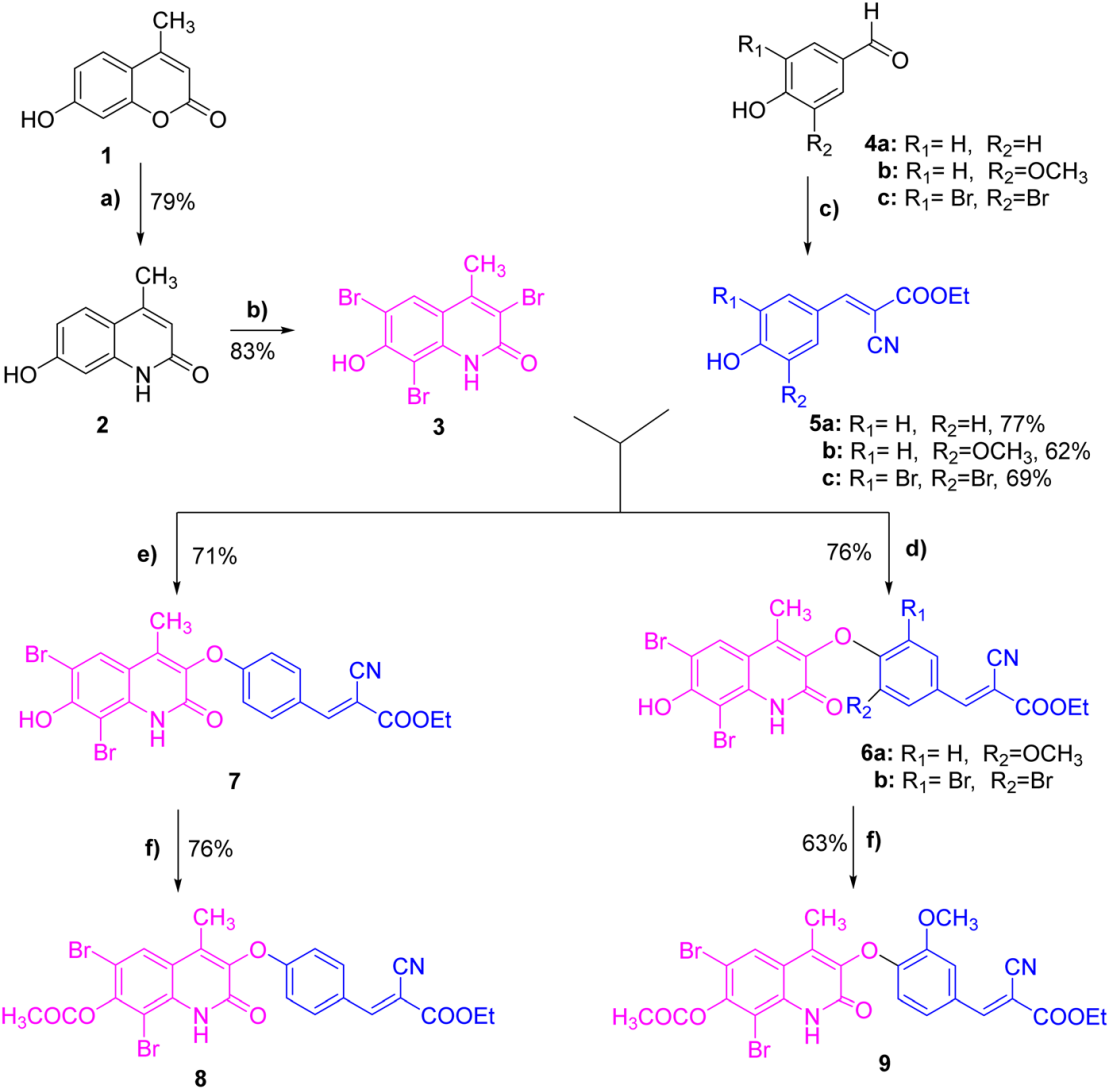
Synthesis of novel azacoumarin–α-cyanocinnamate hybrid compounds (6a, 6b, 7, 8, and 9). Reagents and conditions: a) 33% ammonia, pyridine, EtOH, reflux at 85 °C, 12 h; b) Br_2_, acetic acid, 70 °C, 6 h; c) ethyl cyanoacetate, piperidine (cat.), toluene, reflux at 120 °C, 12 h; d) i – 5b/5c, 2 M NaOH solution; 1 h; ii – 3, reflux at 110 °C, 8 h; e) i – 3, K_2_CO_3_ anhyd, EtOH, 1 h; ii – 5a, reflux at 85 °C, 12 h; f) Ac_2_O, reflux at 150 °C, 6–8 h.

In the present study, a set of novel azacoumarin–α-cyanocinnamate hybrid compounds (6a, 6b, 7, 8, 9) was synthesized following the synthetic strategy illustrated in Scheme 1. The synthesis of the azacoumarin moiety started from the commercially available 7-hydroxy-4-methylcoumarin 1 by reaction with 33% ammonia solution in the presence of pyridine to provide the corresponding azacoumarin analogue 2 in a good yield (79%).^39^ To improve the membrane permeability and metabolic stability of compounds, we envisioned enhancing compound lipophilicity by bromination of the azacoumarin moiety. Bromination of azacoumarin 2 was successfully achieved, following the previously reported procedure,^40^ by reaction with bromine solution under acidic conditions to provide the key intermediate tri-bromo-azacoumarin 3 in a satisfactory yield (83%). On the other hand, the synthesis of the α-cyanocinnamate moiety started from commercially available 4-hydroxy-benzaldehyde 4a or substituted 4-hydroxy-benzaldehyde 4b–c. Thus, the condensation of benzaldehyde 4a–c with ethyl cyanoacetate in the presence of piperidine (cat.) afforded the corresponding ethyl-2-cyano-3-aryl acrylates 5a–c in 62–77% yields.^41^ With the two key moieties in hand, the coupling proceeded under basic conditions to provide the main hybrid structures. In this regard, ethyl (*E*)-2-cyano-3-(4-hydroxyphenyl)-acrylate 5a was reacted with bromo-azacoumarin 3 in the presence of potassium carbonate under reflux at 85 °C to successfully afford compound 7 in considerable yield (71%). Unfortunately, under similar conditions, the reaction of bromo-azacoumarin with the substituted ethyl-2-cyano-3-aryl acrylates 5b–c did not provide the expected products in satisfactory yields. After investigations, we found that applying stronger basic conditions is critical in determining reaction efficiency and product yields. Thus, the reaction of substituted ethyl-2-cyano-3-aryl acrylates 5b–c with bromo-azacoumarin 3 proceeded in 2 M sodium hydroxide solution under reflux at 110 °C to furnish the corresponding azacoumarin–α-cyano cinnamate hybrid compounds 6a and 6b in 61% and 69% yields, respectively. To further explore the role of the hydroxyl group in the azacoumarin moiety, we aimed to acetylate it under acidic conditions. Toward this, compounds 6a and 7 were reacted with acetic anhydride under reflux at 150 °C to provide the corresponding acetylated azacoumarin–α-cyano cinnamate hybrid compounds 8 and 9 in good yields (76% and 63%, respectively). The structure elucidation and purity of all synthesized compounds were assessed by several analytical techniques including elemental analysis, FTIR, NMR (^1^H-NMR and ^13^C-NMR) analysis, and mass spectroscopy.

#### Structural characterization

##### Compound 6a

The FTIR analysis showed a broad absorption at 3375 cm^−1^ for phenolic –OH and 3208 cm^−1^ for NH, along with C=O stretches at 1710–1682 cm^−1^ and aromatic C=C bands at 1608 and 1588 cm^−1^. Signals at 1125–1046 cm^−1^ were attributed to C–O stretches. In the ^1^H NMR spectrum, two methyl resonances were observed at *δ* 1.28–1.31 and 2.22, a methoxy singlet at *δ* 3.82, and a methylene quartet at *δ* 4.26–4.31. Aromatic protons appeared at *δ* 6.86–6.96 and 7.58–7.75, along with singlets at *δ* 7.92 and *δ* 8.20 (olefinic proton). Broad singlets at *δ* 10.56 and *δ* 11.47 confirmed phenolic (OH) and amide (NH) protons. The ^13^C-NMR spectrum revealed carbonyl carbons at *δ* 157.89 and 156.97, oxygenated and bromonated aromatic carbons at *δ* 153.58–155.47, and aromatic/olefinic carbons between *δ* 122.18 and 143.01. The methoxy, methylene, and methyl carbons appeared at *δ* 53.49, 56.00, and 19.73–19.81, respectively.

##### Compound 6b

The FTIR spectra displayed a broad –OH stretch at 3287 cm^−1^, C–H stretches at 2922 and 2826 cm^−1^, a broad C=O band at 1728 cm^−1^, and aromatic C=C bands at 1610 and 1590 cm^−1^. C–O stretching appeared at 1222–1043 cm^−1^. The ^1^H-NMR spectrum showed methyl signals at *δ* 1.28–1.31 and 2.15, a methylene quartet at *δ* 3.70–3.85, and aromatic singlets at *δ* 6.89, 7.78, and 7.96, along with *δ* 8.26 for the olefinic proton. Broad singlets at *δ* 9.88 (NH) and *δ* 11.60 (OH) confirmed amide and phenolic protons. In the ^13^C-NMR spectrum, carbonyl carbons resonated at *δ* 163.22 and 158.80, oxygenated and bromonated aromatic carbons at *δ* 154.21–157.93, and aromatic/olefinic carbons at *δ* 116.74–152.40. Methylene and methyl carbons appeared at *δ* 56.74 and 19.86–19.96.

##### Compound 7

The FTIR spectra exhibited a broad –OH stretch at 3400 cm^−1^, NH at 3200 cm^−1^, and C=O absorptions at 1720–1680 cm^−1^, along with aromatic C=C bands at 1605 and 1585 cm^−1^ and C–O stretches at 1130–1050 cm^−1^. In ^1^H-NMR, a methyl triplet at *δ* 1.28–1.31, a methoxy singlet at *δ* 3.83, and a methylene quartet at *δ* 4.26–4.31 were observed. Aromatic protons appeared at *δ* 6.86–6.98 and 7.93–7.98, with a singlet at *δ* 7.66, and the olefinic proton at *δ* 8.20. Broad singlets at *δ* 10.86 and *δ* 11.49 indicated phenolic (OH) and amide (NH) groups. The ^13^C-NMR spectrum revealed carbonyl signals at *δ* 157.89 and 156.98, oxygenated and bromonated aromatic carbons at *δ* 154.74–155.42, and aromatic/olefinic carbons between *δ* 110.16 and 153.58. Methoxy and methylene carbons appeared at *δ* 63.42 and 62.43, and methyl carbons at *δ* 19.72–19.75.

##### Compound 8a

The FTIR spectra showed aliphatic C–H stretches at 2949 cm^−1^, a sharp cyano absorption at 2220 cm^−1^, carbonyl bands at 1718–1684 cm^−1^, and C=C/C=N stretches at 1610 and 1519 cm^−1^. C–O bands appeared at 1228–1094 cm^−1^, while C–Br stretching bands were observed at 539 and 489 cm^−1^. The ^1^H-NMR spectrum displayed methyl peaks at *δ* 1.21–1.34, 1.77, and 1.92, a methylene quartet at *δ* 4.26–4.36, and aromatic protons at *δ* 6.87–7.00 and 7.36–8.22, with a singlet at *δ* 8.24 (olefinic) and *δ* 8.41 (NH). The ^13^C-NMR spectrum showed carbonyl carbons at *δ* 172.52, 169.28, and 167.41, oxygenated and bromonated aromatic carbons at *δ* 162.27–163.42, and aromatic/olefinic carbons at *δ* 116.07–158.90. Methoxy, methylene, and methyl carbons appeared at *δ* 62.43, 62.89, and 14.46–22.97.

##### Compound 9

The FTIR spectra exhibited a broad –OH stretch at 3383 cm^−1^, NH at 3215 cm^−1^, and broad C=O absorptions at 1713–1685 cm^−1^, along with aromatic C=C bands at 1610 and 1590 cm^−1^ and C–O stretches at 1121–1043 cm^−1^. The ^1^H-NMR spectrum displayed methyl resonances at *δ* 1.22–1.30, 2.20, and 2.31, a methoxy singlet at *δ* 3.38, and a methylene quartet at *δ* 3.84–3.92. Aromatic protons appeared at *δ* 7.33–7.35, 7.72–7.74, and 7.85–7.97, along with singlets at *δ* 7.53 and 7.85. A singlet at *δ* 8.24 corresponded to the olefinic proton, and *δ* 8.41 to the amide NH. The ^13^C-NMR spectrum displayed carbonyl signals at *δ* 169.17, 168.65, and 167.40, oxygenated and bromonated aromatic carbons at *δ* 154.93–162.79, and aromatic/olefinic carbons between *δ* 102.58 and 148.96. Methoxy, methylene, and methyl carbons appeared at *δ* 53.87, 56.45, and 20.68–21.35.

### 2.2 *In vitro* assessment of antiproliferative activity

#### 2.2.1 Evaluation of cytotoxic activity

**Fig. 3:**
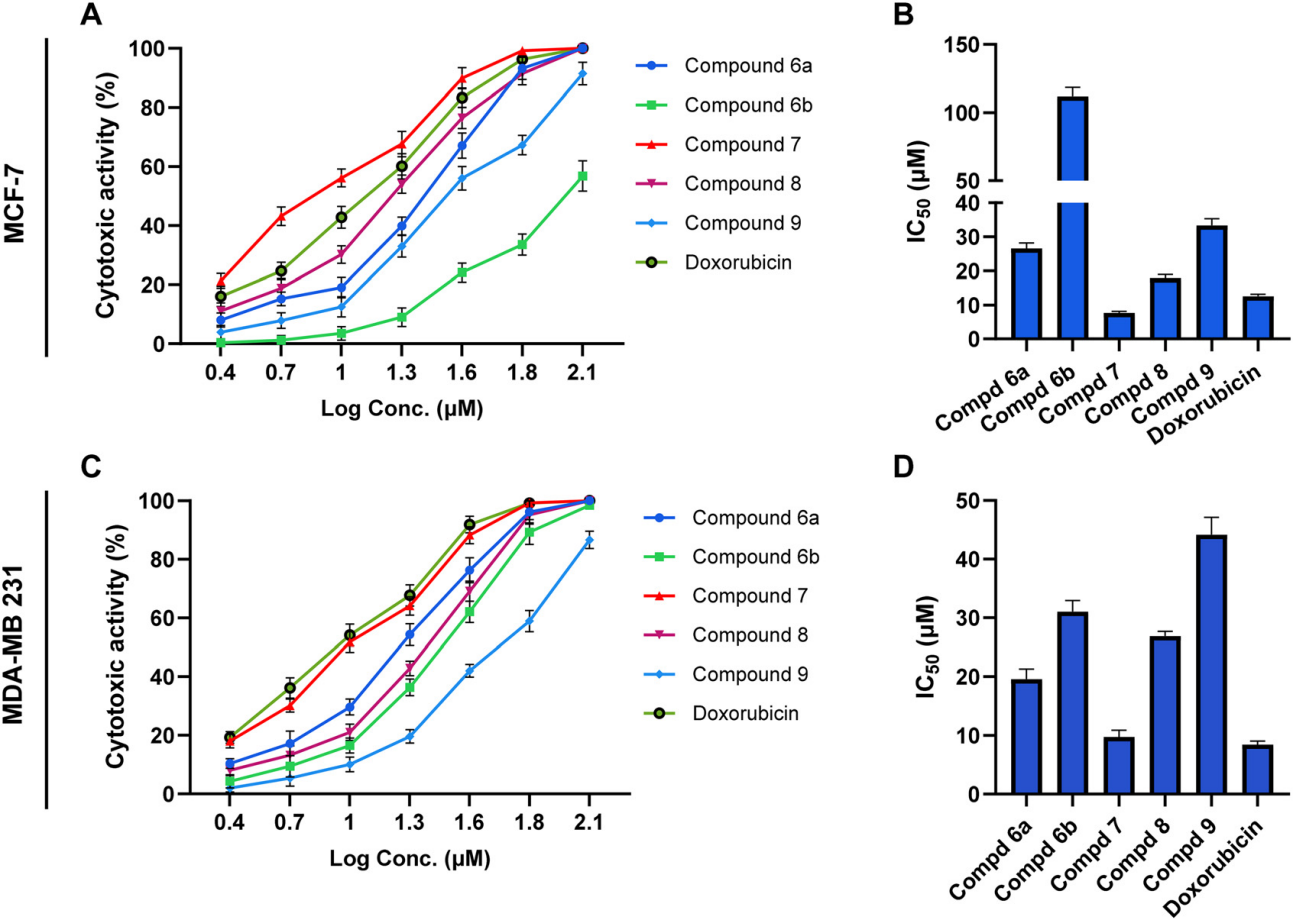
*In vitro* antiproliferative screening of the synthesized compounds (6a, 6b, 7, 8, and 9) toward human breast cancer MCF-7 (A and B) and MDA-MB 231 (C and D) cells. Representative dose–response cytotoxic activity of synthesized compounds toward MCF-7 (A) and MDA-MB 231 (C) cells. Representative IC_50_ values for the antiproliferative activity of synthesized compounds toward MCF-7 (B) and MDA-MB 231 (D) cell viability. Data are presented as mean ± SD from, with *n* = 3 experiments.

We first assessed the antiproliferative activity of the synthesized compounds (6a, 6b, 7, 8, and 9) against the cellular viability of MCF-7 and MDA-MB 231 utilizing the well-known MTT assay. In our examinations, doxorubicin was applied as a reference anticancer drug. The examined compounds were assessed at different concentrations, and the IC_50_ values were obtained from the inhibition curve. As indicated in Fig. 3, the synthesized azacoumarin–α-cyanocinnamate hybrids exhibited considerable cytotoxic activity toward the MCF-7 cells in a dose-dependent manner. The evaluation of the structure–activity relationship indicated that the substitution at the phenyl ring in the cyano-cinnamate moiety plays a crucial role in the cytotoxic activity of this class of hybrid compounds. As shown in Fig. 3, compound 6a, which displayed a methoxy-substitution at position-3 in the cyano-cinnamate moiety, exhibited reasonable antiproliferative activity with IC_50_ = 26.57 ± 1.56 μM. The substitution at positions 3 and 5 of the phenyl ring led to significant attenuation in the antiproliferative activity, as shown in compound 6b with IC_50_ = 112 ± 6.57 μM. Interestingly, compound 7, which displayed an unsubstituted phenyl ring at the cyano-cinnamate moiety, demonstrated a substantial antiproliferative activity with IC_50_ = 7.653 ± 0.45 μM. These findings indicate that the substitution at the phenyl ring impairs the cytotoxic activity of the hybrid structure, likely by steric hindrance, leading to loss of binding with the target protein(s). Further, our investigations revealed that the hydroxyl group at the azacoumarin moiety also plays a considerable role in the activity of the hybrid structure. Acetylation of the hydroxyl group in compound 7 resulted in a significant reduction in the cytotoxic activity, as indicated in compound 8 with IC_50_ = 17.84 ± 1.05 μM, with a 2.3-fold reduction. Similarly, acetylation of the hydroxyl group in compound 6a led to an amelioration of the activity as shown in compound 9 with IC_50_ = 33.35 ± 1.96 μM, with a 1.25-fold reduction (Table S1). These results further indicate that the hydroxyl group may participate in the cytotoxic activity of this class of hybrid structure, likely by forming H-bondings with targeted protein(s). To further verify these observations, the antiproliferative activity of the compounds was assessed against the triple-negative breast cancer cell line MDA-MB-231. The trend of activity across the series was generally consistent with that observed in MCF-7 cells, with compound 7 again showing the most potent effect (IC_50_ = 9.7 ± 1.15 μM), approaching the activity of doxorubicin (IC_50_ = 8.4 ± 0.61 μM). Compound 6a demonstrated moderate activity (IC_50_ = 19.6 ± 1.72 μM), while the 3,5-disubstituted phenyl ring (6b) significantly reduced potency (IC_50_ = 31.1 ± 1.87 μM). Acetylated derivatives 8 and 9 showed decreased activity (IC_50_ = 26.9 ± 0.79 μM and 44.2 ± 2.96 μM, respectively), supporting the importance of the free hydroxyl group at the azacoumarin moiety for optimal activity. Notably, most compounds exhibited enhanced potency in MDA-MB-231 cells compared to MCF-7, particularly compound 6b, which showed an ∼3.6-fold improvement in cytotoxicity, suggesting potential differential sensitivity of triple-negative breast cancer cells to this scaffold (Table S1). Our results are in alignment with several reported studies, which showed that the azacoumarin–α-cyanocinnamate hybrids display substantial antiproliferative potential toward various cancer cells.^24,42^ Among the tested compounds, compound 7 demonstrated the most antiproliferative activity against the viability of MCF-7 and MDA-MB 231 cells, with an IC_50_ value compared to that of the reference doxorubicin (IC_50_ = 12.55 ± 0.61 μM and 8.43 ± 0.36 μM toward MCF-7 and MDA-MB 231 cells, respectively). To further explore the selectivity of compound 7 toward cancer cells, we examined its cytotoxicity toward MCF-10A cells and compared it to that of doxorubicin. Our results revealed that compound 7 displays a cytotoxic activity at considerably high concentrations, with IC_50_ = 52.02 ± 1.76 μM, as compared to the anticancer doxorubicin drug with IC_50_ = 16.19 ± 0.55 μM (Fig. S1). These results indicate that compound 7 possesses significant selectivity toward breast cancer cells with a selectivity index ranging from 5.3 to 6.8, compared to doxorubicin, which displays an SI of 1.3. Together, our study supports the promising antiproliferative activity of the presented azacoumarin–α-cyanocinnamate hybrids, especially compound 7, which demonstrated the most potent activity with a high selectivity index toward MCF-7 and MDA-MB 231 cells, exceeding that of the anticancer doxorubicin.

#### 2.2.2 Assessment of cell cycle analysis

**Fig. 4:**
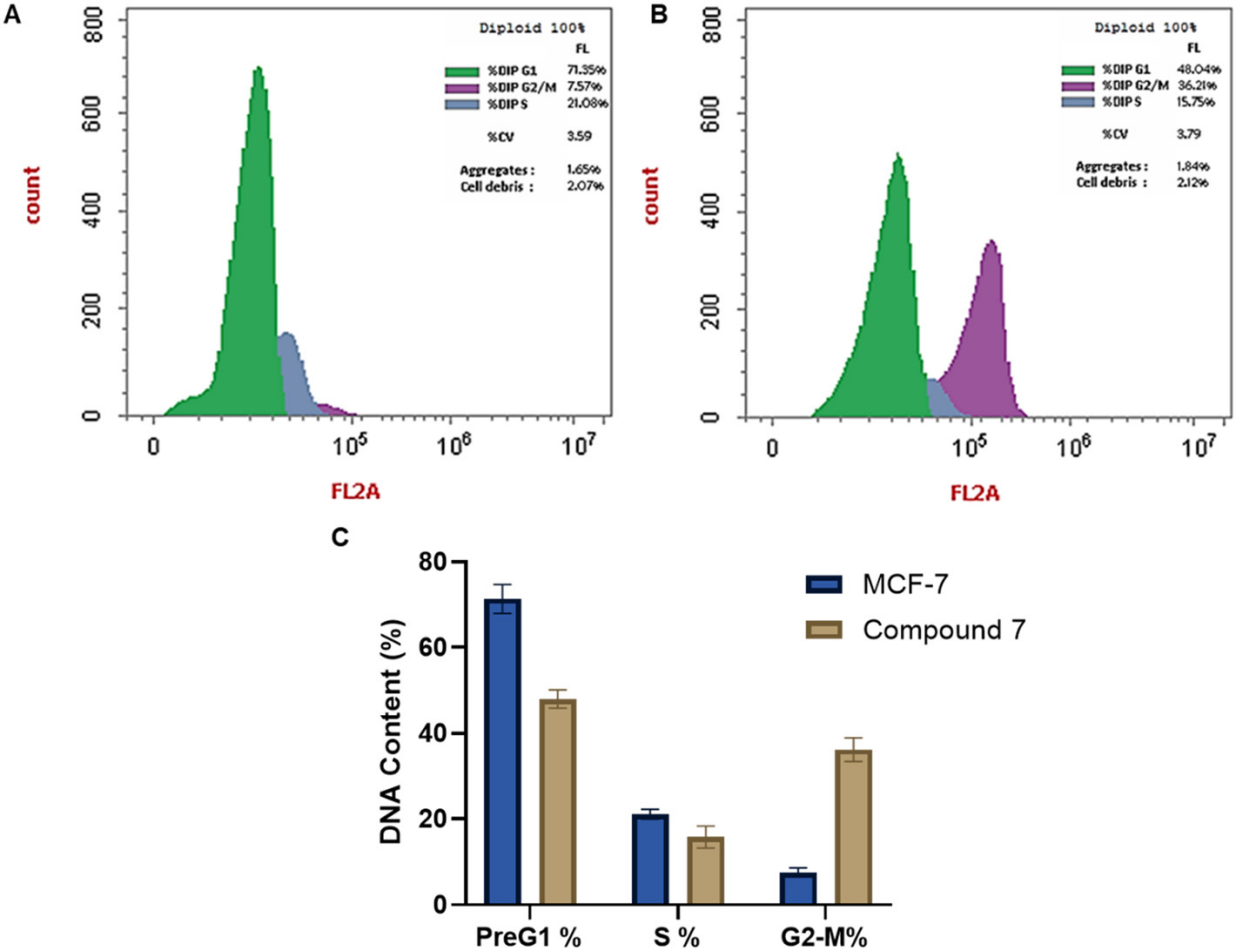
Representative flow cytometric analysis of the cell cycle phases of MCF-7 cells showing the influence of compound 7 at 7.65 μM concentration after incubation for 24 h. A) DNA content distribution in the untreated MCF-7 cells. B) DNA content distribution in MCF-7 cells after treatment with compound 7. C) Graphical analysis of the DNA content distribution in the different cell cycle phases of MCF-7 cells showing the effect of compound 7 treatment on cell cycle dynamics.

Inducing cell cycle arrest is a central strategy in anticancer therapy, particularly for compounds targeting rapidly dividing tumor cells. To investigate the antiproliferative mechanism of compound 7, flow cytometry analysis was performed on MCF-7 cells treated with its IC_50_ concentration (7.653 μM) for 24 hours. As shown in Fig. 4, compound 7 induced a significant G_2_/M phase arrest, with 36.21% of cells accumulating in this phase compared to only 7.57% in the untreated control. This arrest was accompanied by a reduction in S-phase cells (from 21.08% in the control to 15.75%) and a notable shift in pre-G1 subpopulation (from 71.35% in the control to 48.04%), suggesting that compound 7 interferes with cell cycle progression after DNA synthesis and before mitotic entry. These results indicate that the hybrid structural features of compound 7 may underlie this activity. Align with our findings, previous studies have demonstrated that coumarin-hybrid derivatives can induce G_2_/M phase arrest in breast cancer cells.^43,44^ Taken together, the observed G_2_/M arrest confirms that compound 7 not only exerts cytotoxicity but also disrupts critical checkpoints in the cell cycle, possibly through modulation of mitotic regulators.

#### 2.2.3 Annexin V-FITC/PI screening

**Fig. 5:**
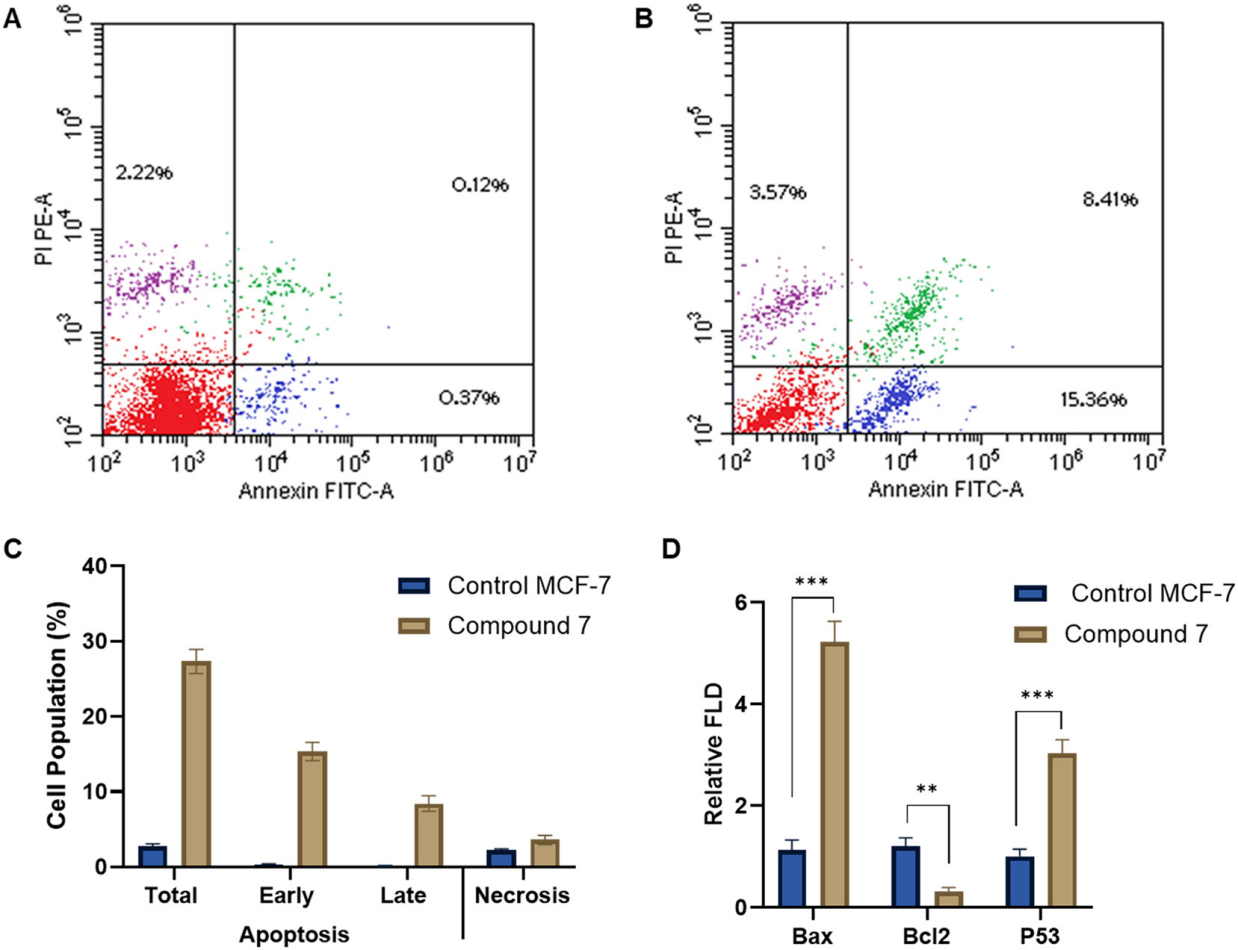
Representative flow cytometric and qRT-PCR analysis showing the impact of compound 7 (7.65 μM) on triggering programmed cell death in MCF-7 cells after incubation for 24 h. A) The flow cytometric annexin V-FITC/PI shows the cell population in untreated MCF-7 cells. B) The flow cytometric annexin V-FITC/PI shows the cell population in MCF-7 cells treated with compound 7. C) Effect of compound 7 on triggering apoptosis and necrosis in MCF-7 cells. D) Effect of compound 7 treatment on the relative expression of intrinsic apoptotic p53, Bax, and Bcl2 genes in MCF-7 cells.

In cancer therapy, inducing apoptosis in tumor cells is a primary strategy to inhibit cancer progression. To evaluate the apoptotic effects of compound 7 on MCF-7 breast cancer cells, we employed the annexin V-FITC/PI assay. In this regard, treatment of MCF-7 cells with compound 7 at its IC_50_ concentration for 24 hours resulted in a significant increase in apoptotic cell populations compared to the untreated control group. As shown in Fig. 5, early apoptotic cells increased from 0.37% in the control to 15.36%, and late apoptotic cells rose from 0.12% to 8.41%. The total apoptosis (early and late) accounted for 27.34% of the cell population post-treatment, a substantial elevation from the 2.71% observed in the control group. A minor change in the necrotic cell population was observed, increasing slightly from 2.22% in the control to 3.57% in the treated cells. These results suggest that compound 7 induces apoptosis as the predominant mode of cell death in MCF-7 cells. The induction of apoptosis by compound 7 aligns closely with the observed G_2_/M cell cycle arrest, suggesting a sequential mechanism of action. Following cell cycle blockade, MCF-7 cells likely undergo intrinsic programmed cell death due to the inability to resolve checkpoint arrest. Our findings are in agreement with a recent report, which showed that a quinoline–cinnamate hybrid exhibits potential antiproliferative activity by apoptosis induction.^42^ To further understand the mechanism by which compound 7 induces apoptosis, we assessed the expression of key regulatory proteins involved in the intrinsic apoptotic pathway, including Bax, Bcl-2, and p53, using qRT-PCR analysis. Our analysis revealed that treatment of MCF-7 cells with compound 7 at its IC_50_ concentration (7.653 μM) for 24 hours significantly altered the expression of these markers. As shown in Fig. 5, Bax levels increased 5.22-fold, while Bcl-2 expression was reduced to 0.312-fold relative to the untreated control. Moreover, p53 expression was upregulated 3.03-fold. Our data indicate a clear activation of mitochondrial-mediated apoptosis, characterized by an increased Bax/Bcl-2 ratio and p53 activation. These molecular events align with our previous findings of G_2_/M cell cycle arrest and annexin V-FITC/PI-based apoptosis induction, supporting a sequential mechanism in which compound 7 triggers DNA damage, activates p53, and drives intrinsic apoptotic signaling. Collectively, our results support that compound 7 exerts its antiproliferative effect through a dual mechanism involving G_2_/M phase arrest and modulation of key upstream regulators of apoptosis.

#### 2.2.4 Evaluation of FRAP antioxidant activity

**Fig. 6:**
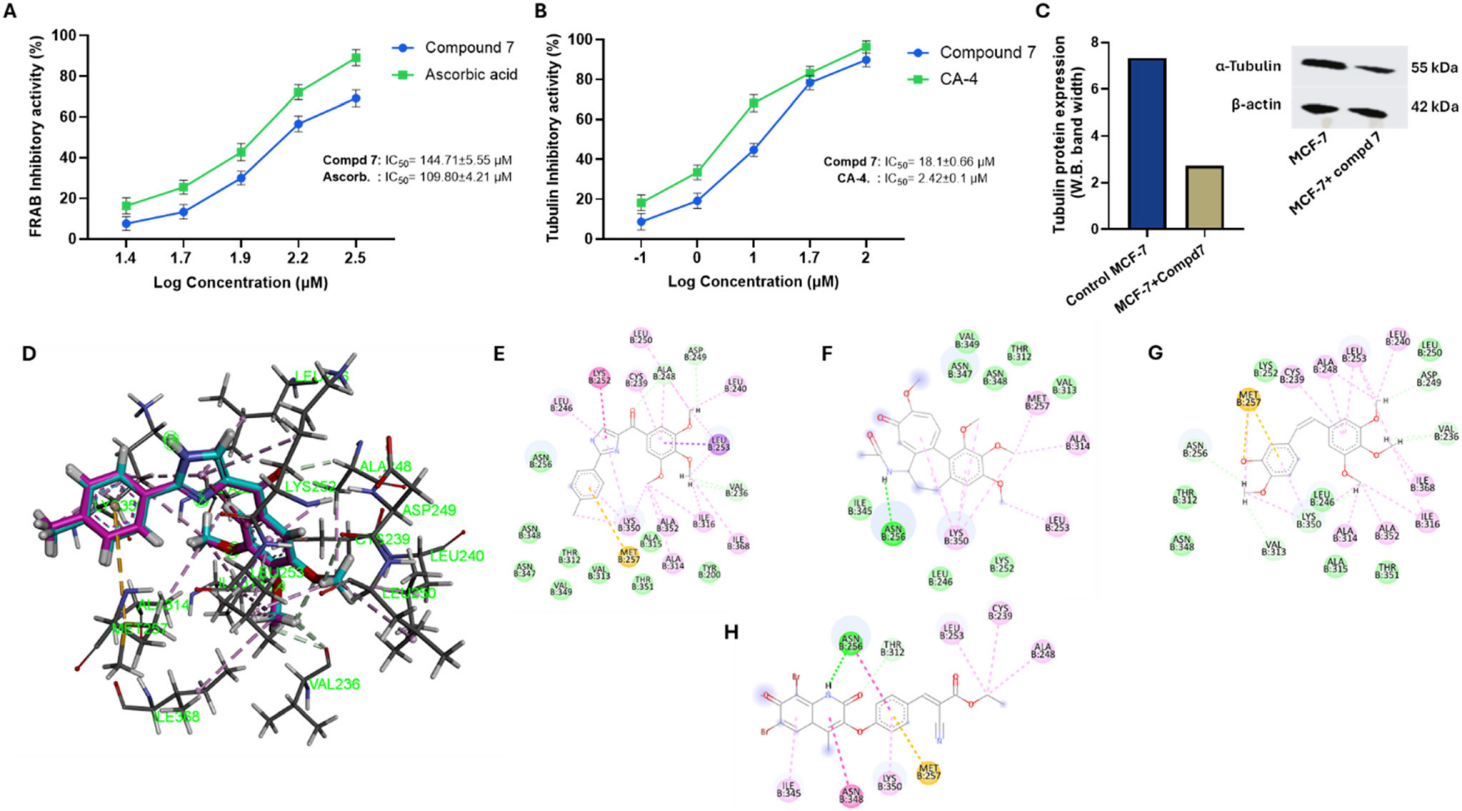
Representative graphs for the effect of compound 7 on the tubulin polymerization activity and its antioxidant activity. (A) Representative dose-dependent antioxidant effect of compound 7 and ascorbic acid on FRAB assay. (B) Representative dose-dependent inhibitory effect of compound 7 and combretastatin A-4 on tubulin polymerization activity. Data are presented as mean ± SD, *n* = 3 experiments. (C) Effect of compound 7 (at its IC_50_ value) on the expression of tubulin protein in MCF-7 cells. (D–H) Binding mode and interactions of ABI-274 (D and E), CA-4 (F), colchicine (G), and compound 7 (H) toward the binding pocket of tubulin protein (PDB: *6pc4*).

To further explore the antiproliferative potential of compound 7, we assessed its antioxidant capacity by measuring its ability to reduce Fe^3+^ ions to Fe^2+^ ions under acidic conditions using the FRAP assay. Our results indicated that compound 7 displays a substantial and dose-dependent antioxidant activity toward FRAP assay. As shown in Fig. 6, compound 7 demonstrated a remarkable ferric-reducing ability, with an IC_50_ value of 144.71 ± 5.55 μM, compared to ascorbic acid, which exhibited an IC_50_ of 109.80 ± 4.21 μM. The observed antioxidant potential of compound 7 further impacts its antiproliferative activity by reducing ROS levels leading to modulation of oxidative stress, thereby promoting apoptosis. The antioxidant activity of compound 7 may be attributed to its coumarin–cinnamate hybrid structure. Several reports showed that both coumarin- and cinnamic-based compounds exhibit substantial antioxidant activity by scavenging free radicals and inhibiting lipid peroxidation.^45–47^ Together, the significant antioxidant activity of compound 7 further highlights its potential as an antiproliferative agent by multitargeting oxidative stress, cell cycle, and cell proliferation.

#### 2.2.5 Evaluation of tubulin polymerization activity

To further explore the mechanism underlying the antiproliferative effect of compound 7, we examined its influence on tubulin polymerization activity. Toward this, the purified tubulin protein was treated with varying concentrations of compound 7, and the enzyme activity was assessed using a microplate reader. In our study, combretastatin A-4 (CA-4) was utilized as a reference control. As shown in Fig. 6, compound 7 exhibited considerable inhibitory activity toward tubulin activity in a dose-dependent manner. The assessment of the IC_50_ value revealed that compound 7 led to a significant inhibition in tubulin polymerization activity with IC_50_ = 18.74 ± 0.66 μM, as compared to CA-4 with an IC_50_ of 2.24 ± 0.1 μM. Additionally, western blot analysis was conducted to evaluate the expression levels of α-tubulin in MCF-7 cells treated with compound 7 (at its IC_50_ = 7.653 μM) compared to untreated control cells. Our results indicated that treating MCF-7 cells with compound 7 significantly reduced (2.7-fold) the expression of α-tubulin protein, as compared to untreated control cells (Fig. 6). Disruption of tubulin polymerization is a well-known mechanism of anticancer agents, which induces mitotic arrest and apoptosis in cancer cells.^48^ Our results further provide mechanistic insight into the antiproliferative activity of compound 7 by destabilizing microtubule dynamics, leading to cell cycle arrest at the G_2_/M phase and triggering apoptotic signaling cascades. Previous studies showed that coumarin-cinnamate-based compounds possess the ability to target tubulin polymerization and induce apoptosis in cancer cells.^24,42,49,50^ These findings were further affirmed by the molecular docking study of compound 7 toward the tubulin pocket, as compared to the original ligand ABI-274, combretastatin A-4, and colchicine. As depicted in Fig. 6, the co-crystallized ligand ABI-274 was first redocked into the binding site of tubulin (PDB: *6PC4*). The resulting RMSD of 0.12 Å between the docked and crystallographic pose confirmed the accuracy of the docking parameters and successful reproduction of the native binding mode (Fig. 6D and E). ABI-274 exhibited a binding affinity of −8.92 kcal mol^−1^ and interacted with key residues including Lys252, Leu253, and Met257 through π–amide stacking, π–sigma, and π–sulfur interactions (Table S2). These interacting residues lie within the colchicine-binding pocket, which is crucial for microtubule destabilization and anticancer activity. Notably, Met257 appears consistently among high-affinity binders, highlighting its role as a critical interaction point for ligand anchoring. Similarly, CA-4 displayed a comparable binding energy of −8.25 kcal mol^−1^, forming a key π–sulfur interaction with Met257, mimicking part of ABI-274's binding mode (Fig. 6F). In contrast, colchicine showed a relatively weaker binding affinity of −6.09 kcal mol^−1^, forming a hydrogen bond with Asn256, a residue proximal to the critical core of the pocket (Fig. 6G). Interestingly, compound 7 displayed a significant binding affinity of −7.58 kcal mol^−1^ into the colchicine-binding pocket, exceeding that of colchicine (Table S2). Notably, compound 7 maintained interaction with Met257 *via* a π–sulfur interaction but also formed hydrogen bonds with Asn256 and amide–π interactions with both Asn256 and Asn348 (Fig. 6H). These findings indicate that compound 7 occupies the colchicine-binding cavity, preserving essential interactions that are recognized as critical for inhibition of tubulin polymerization. Together, our findings indicate that compound 7 exerts a multi-targeted antiproliferative activity toward MCF-7 cells by microtubule destabilization, cell cycle arrest, and activation of the intrinsic apoptotic pathway.

#### 2.2.6 Evaluation of the anti-inflammatory activity (COX-1 and COX-2)

**Fig. 7:**
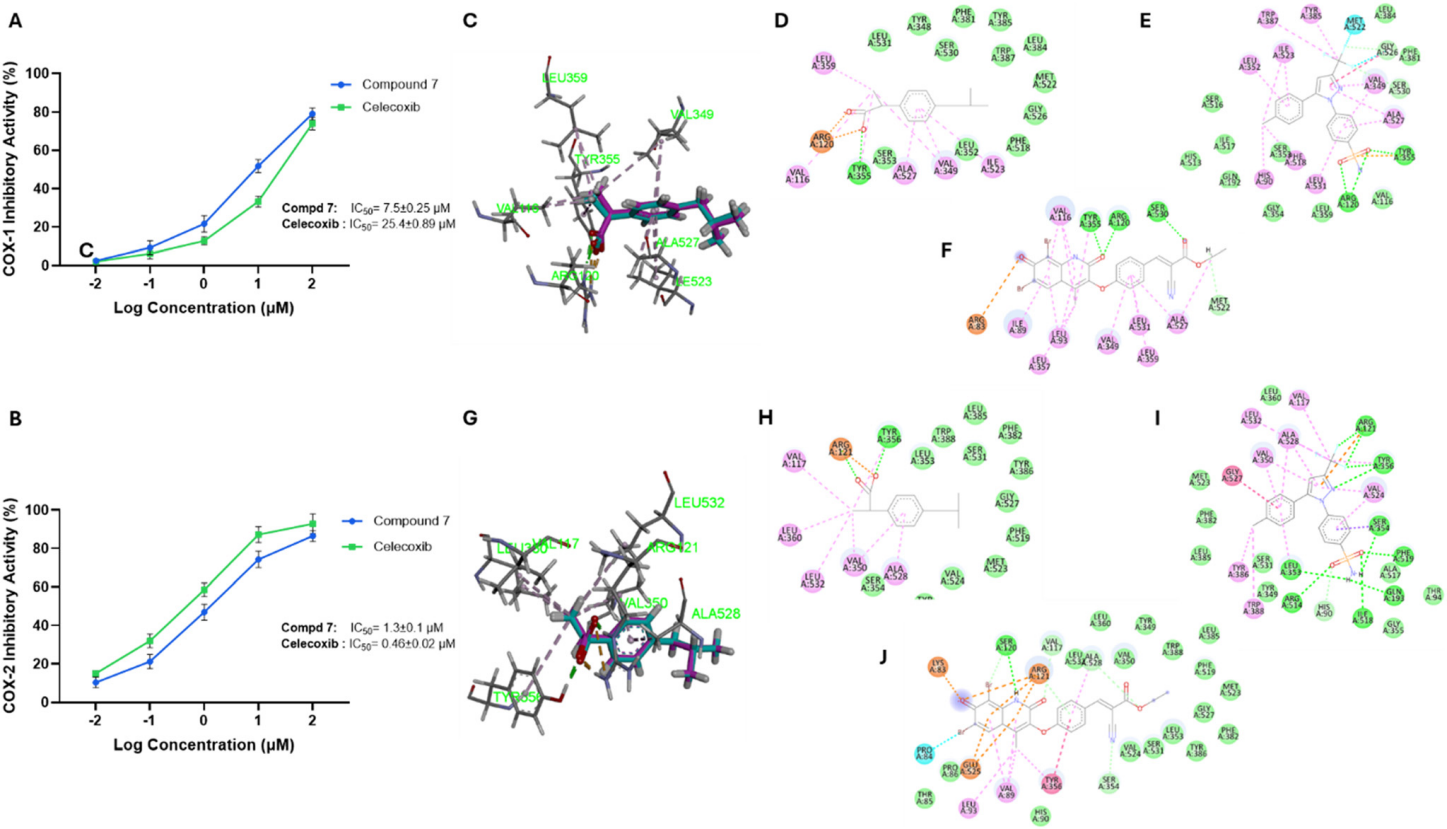
Representative graphs for the assessment of the inhibitory activity and binding affinity of compound 7 toward the cyclooxygenases (COX1 and COX2). (A and B) Representative dose–response inhibitory activity of compound 7 toward COX1 (A) and COX2 activity (B). Data are presented as mean ± SD, with *n* = 3 experiments. (C–F) Binding mode and interactions of ibuprofen (C and D), celecoxib (E), and compound 7 (F) toward the binding pocket of COX-1 protein (PDB: *1eqg*). (G-K) Binding mode and interactions of ibuprofen (G and H), celecoxib (I), and compound 7 (J) toward the binding pocket of COX-2 protein (PDB: *4ph9*).

To evaluate the anti-inflammatory potential of compound 7 and its ability to modulate key inflammatory pathways, we assessed its inhibitory effect on cyclooxygenase-1&2 (COX-1&2) enzymes. These enzymes catalyze the conversion of arachidonic acid to prostaglandins, which are key mediators of inflammation, pain, and tumorigenesis. COX-2, in particular, is upregulated in many cancers and supports tumor proliferation, angiogenesis, and immune evasion.^51^ As indicated in Fig. 7, compound 7 demonstrated a potential and dose-dependent inhibitory activity toward both COX isoforms. The assessment of IC_50_ revealed that compound 7 exhibits an IC_50_ of 7.492 ± 0.25 μM toward COX-1, while the IC_50_ toward COX-2 was 1.264 ± 0.1 μM, yielding a selectivity index (SI) of 5.93. On the other hand, celecoxib exhibited IC_50_ = 25.39 ± 0.85 μM toward COX-1 and IC_50_ = 0.463 ± 0.02 μM toward COX-2, with a SI of 54.83, confirming its high COX-2 selectivity. While compound 7 showed a considerable selectivity index, it displayed a substantial inhibitory activity toward COX-2. To further explore the selectivity of compound 7 and celecoxib toward COX-1&2, we extended our investigations to perform a molecular docking study. Initially, we redocked ibuprofen (co-crystallized ligand) into the COX-1 binding site (PDB: *1eqg*) to validate the docking protocol. Our analysis revealed that the redocked ibuprofen exhibits an RMSD of 0.33 Å with a considerable binding affinity of −7.93 kcal mol^−1^, indicating the reliability of the docking parameters (Table S2, Fig. 7C). Further, the redocked ibuprofen showed interactions with Arg120 through ionic and salt-bridge interactions, and formed a hydrogen bond with Tyr355, consistent with its known engagement in the active site (Fig. 7D). On the other hand, celecoxib exhibited a binding affinity of −3.97 kcal mol^−1^ and displayed interactions with Arg120, Tyr385, and Gly526 *via* hydrogen bonds, amide–π stacking, and a π–sulfur interaction, suggesting partial compatibility with the COX-1 binding pocket but with lower stability compared to the native ligand (Fig. 7E). Compound 7 demonstrated a considerable binding affinity of −6.28 kcal mol^−1^ toward COX-1 and displayed a critical interaction with Arg120, a conserved anchor residue involved in COX selectivity and inhibition. It also formed additional hydrogen bonds with Tyr385 and Ser530, as well as an ionic interaction with Arg83, contributing to its stabilization within the pocket (Fig. 7F). Regarding the COX-2 enzyme, the redocking of ibuprofen achieved an RMSD of 0.14 Å and displayed a binding energy of −7.47 kcal mol^−1^, validating the docking protocol (Table S2, Fig. 7G). Further, ibuprofen exhibited critical interactions with Arg121 (through hydrogen bonding and a salt bridge) and Tyr356, indicating structural overlap with the crystallographic conformation (Fig. 7H). Aligned with its known selectivity, celecoxib displayed a substantial binding affinity (−8.31 kcal mol^−1^) and formed an interaction network within the COX-2 binding pocket, including Arg121, Tyr356, Ser354, Gln193, Arg514, and Leu353. Additionally, π–sigma and amide–π interactions with Phe519 and Gly527 contributed to its high binding affinity and stability within the enlarged active site of COX-2 (Fig. 7I). On the other hand, compound 7 exhibited a binding energy of −7.09 kcal mol^−1^ and displayed interactions with the core anchoring residues Ser120, Arg121, and Tyr356 but also established a π–cation interaction with Lys83, a π–anion interaction with Glu525, and π–π stacking with Tyr356 (Fig. 7J). Aligning with our *in vitro* analysis, these results imply that compound 7 possesses considerable affinity and selectivity toward the COX-2 enzyme (Table S2). COX-2 has been implicated in promoting resistance to apoptosis and enhancing microtubule stability *via* PGE_2_-mediated pathways. Thus, its inhibition by compound 7 may reduce pro-inflammatory prostaglandins, induce apoptosis, and disturb tubulin activity.^52,53^ Moreover, COX-2 inhibition is known to modulate the intracellular redox balance, supporting the antioxidant potential of compound 7, as confirmed by the FRAP assay. These features may be associated with the hybrid azacoumarin–cinnamate scaffold of compound 7. The presence of electron-donating hydroxyl groups, conjugated double bonds, and a coumarin moiety in compound 7 likely enhances both radical stability and enzyme binding affinity.^54,55^ Together, these results support the multi-targeted activities of compound 7 through modulation of oxidative stress, COX-2-induced inflammation, and pro-apoptotic activities.

### 2.3 *In vivo* evaluation of therapeutic potential in the EAC-induced animal model

#### 2.3.1 Assessment of lethal dose (LD)

To evaluate the safety profile and determine the maximum tolerated dose (MTD) of compound 7, an acute toxicity study was performed in mice. Compound 7 was administered intraperitoneally at escalating doses ranging from 1 to 200 mg kg^−1^, and animals were monitored over a 48 hour period for signs of toxicity, behavioral changes, and mortality. Our results revealed that animals receiving doses up to 100 mg kg^−1^ exhibited normal behavior, physical activity, grooming, feeding, and excretion patterns, with no observable signs of toxicity or mortality. However, at a dose of 200 mg kg^−1^, animals exhibited pronounced signs including lethargy, decreased locomotor activity, signs of exhaustion, and rapid progression to mortality. Based on these observations, the maximum tolerated dose (LD_0_) of compound 7 was determined to be 100 mg kg^−1^, while the approximate lethal dose (LD_100_) lies at 200 mg kg^−1^. Based on these findings, all subsequent pharmacological, biochemical, and histological assessments in this study were conducted at doses well below the LD_0_, ensuring that observed effects reflect therapeutic potential rather than toxicity.

#### 2.3.2 Assessment of effective dose

**Fig. 8:**
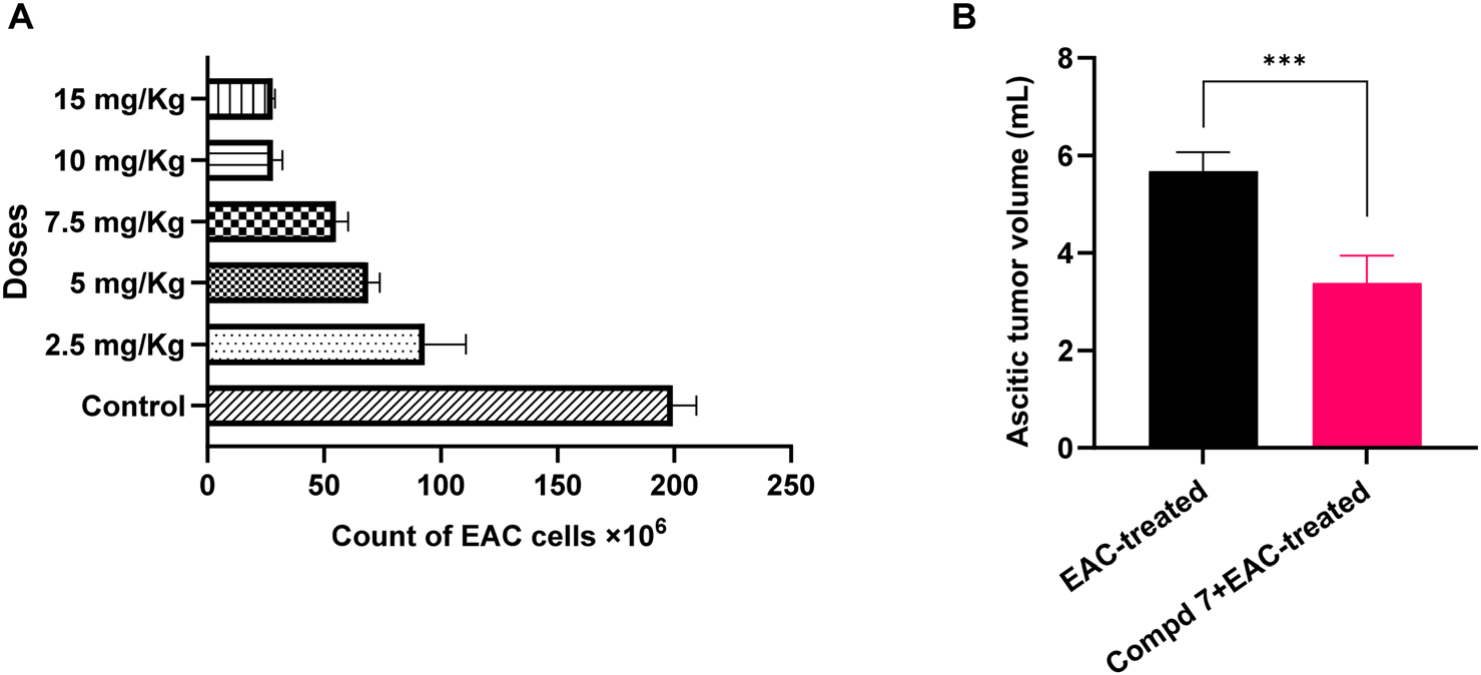
Effect of compound 7 on the viability of EAC cells and ascitic tumor volume in EAC-treated mice. A) Effect of compound 7 at different doses on the EAC cell count. B) Effect of compound 7 at 10 mg kg^−1^ dose on the ascitic tumor volume in the EAC-treated model. Data are presented as mean ± SE, with *n* = 6. The statistical analysis was conducted using one-way ANOVA and considered significant at *p* ≤ 0.05 (****p ≤ 0.0001, ***p ≤ 0.001, **p ≤ 0.01, and *p ≤ 0.05).

The assessment of effective dose is a critical step in drug development, linking pharmacological activity to therapeutic efficacy.^56^ To determine the optimal therapeutic dose of compound 7, we evaluated its *in vivo* ability to reduce the viability of EAC cells. Toward this, EAC-bearing mice were treated intraperitoneally with compound 7 at varying doses (2.5, 5, 7.5, 10, and 15 mg kg^−1^) every two days for a total of 10 days. At the end of the treatment period, total viable EAC cells were quantified using the trypan blue exclusion assay. As shown in Fig. 8, compound 7 displayed a dose-dependent reduction in the EAC cell count, which was statistically significant across all treatment groups (*p* < 0.0001, one-way ANOVA). In the positive (untreated) EAC control group, the total viable EAC cell count was 199.33 ± 4.15 × 10^6^ cells. Treatment with 2.5 mg kg^−1^ reduced the viable count to 93.15 ± 7.19 × 10^6^ cells (53.27% reduction), while 5 mg kg^−1^ and 7.5 mg kg^−1^ further lowered the count to 68.95 ± 2.00 × 10^6^ (65.41%) and 55.10 ± 2.15 × 10^6^ (72.36%), respectively. A substantial reduction was observed at 10 mg kg^−1^, where cell viability dropped to 28.06 ± 1.69 × 10^6^ cells, reflecting an 85.92% reduction. The 15 mg kg^−1^ dose produced a nearly identical reduction (27.98 ± 0.39 × 10^6^ cells, 85.96%), suggesting a plateau effect beyond 10 mg kg^−1^. Further, the examination of tumor volume revealed that the therapeutic group treated with 10 mg kg^−1^ displays a significantly reduced average ascitic tumor volume (3.11 ± 0.45 mL), compared to 5.68 ± 0.15 mL in the untreated EAC group, with a 45.25% reduction (*p* < 0.0001). These findings imply that compound 7 at the selected dose not only limits cell survival but may also suppress tumor growth kinetics *via* antiproliferative or pro-apoptotic mechanisms, supporting our previous *in vitro* studies. Collectively, these data suggest that compound 7 displays potent antitumor activity in a dose-dependent manner, with 10 mg kg^−1^ emerging as the optimal effective dose for subsequent pharmacological, biochemical, and histological studies.

#### 2.3.3 Assessment of serum liver and kidney function markers

**Fig. 9:**
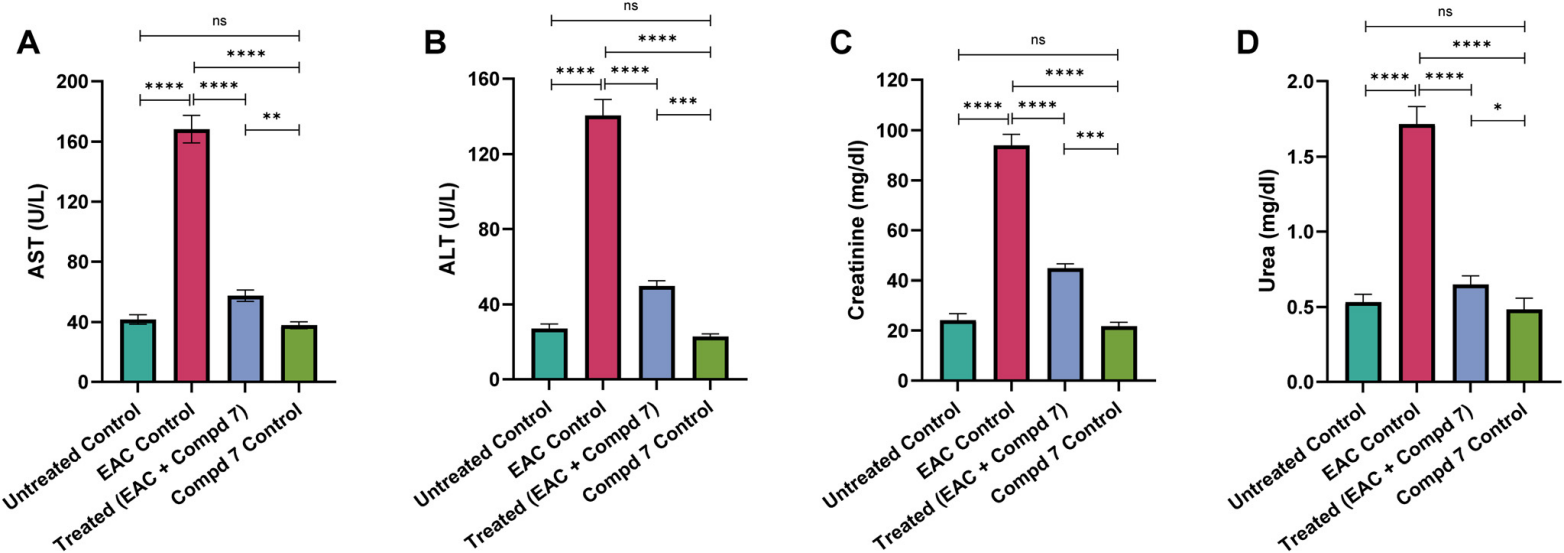
Effect of compound 7 treatment on the expression levels of key markers for liver and kidney function ((A) ALT, (B) AST, (C) creatinine, and (D) urea) among experimental groups. Data are presented as mean ± SE, with *n* = 6. The statistical analysis was conducted using one-way ANOVA and two-way ANOVA and considered significant at *p* ≤ 0.05 (****p ≤ 0.0001, ***p ≤ 0.001, **p ≤ 0.01, and *p ≤ 0.05).

To evaluate the hepatorenal toxicity induced by EAC and the potential protective role of compound 7, we assessed liver and kidney function by measuring serum levels of alanine aminotransferase (ALT) and aspartate aminotransferase (AST), two key hepatic enzymes released during hepatocellular injury, as well as serum creatinine and urea, established indicators of renal function. As shown in Fig. 9, the EAC control group exhibited a substantial increase in liver enzyme levels, with ALT rising to 140.66 ± 3.45 U L^−1^ and AST to 168.33 ± 3.7 U L^−1^, compared to the untreated control group, which showed normal values (ALT: 27.16 ± 0.94 U L^−1^; AST: 41.66 ± 1.3 U L^−1^, *p* < 0.0001). Similarly, serum creatinine and urea levels were significantly elevated in the EAC group (1.7 ± 0.04 mg dL^−1^ and 94 ± 1.78 mg dL^−1^, respectively) compared to controls (0.5 ± 0.02 mg dL^−1^ and 24.16 ± 1.07 mg dL^−1^, *p* < 0.0001), indicating substantial hepatorenal impairment. Treatment with compound 7 significantly improved these parameters. Liver enzymes were reduced to near-normal levels (ALT: 49.83 ± 1.16 U L^−1^; AST: 57.5 ± 1.56 U L^−1^) and kidney function markers showed marked recovery (creatinine: 0.6 ± 0.02 mg dL^−1^; urea: 44.83 ± 0.7 mg dL^−1^), with all differences statistically significant *versus* the EAC group (*p* < 0.0001). These findings indicate the potential protective effect of compound 7 against EAC-induced hepatorenal dysfunction and highlight its potential therapeutic benefit in managing cancer-related metabolic disorders. Notably, the compound 7-control group showed no signs of hepatic or renal toxicity, with values comparable to the untreated control group (ALT: 22.83 ± 0.6 U L^−1^; AST: 37.83 ± 0.94 U L^−1^; creatinine: 0.5 ± 0.03 mg dL^−1^; urea: 21.83 ± 0.6 mg dL^−1^), confirming the safety of compound 7 in non-tumor-bearing animals. The liver and kidneys are primary targets of systemic toxicity in cancer and chemotherapy-induced organ dysfunction.^57^ Our results indicated that the EAC-bearing mice displayed significantly elevated levels of both hepatic and renal markers, reflecting significant tissue damage and metabolic disturbance induced by EAC treatment.^58^ These findings are consistent with prior studies reporting that EAC progression is accompanied by hepatotoxicity and nephrotoxicity due to oxidative stress, inflammatory infiltration, and metabolic reprogramming.^59,60^ Treatment with compound 7, however, reversed these pathological changes, significantly normalizing ALT, AST, creatinine, and urea levels. This improvement is likely attributed to the compound's antioxidant, anti-inflammatory, and cytoprotective effects, as supported by previously described *in vitro* studies. The lack of alteration in the compound 7 control group provides strong evidence for the non-toxic nature of compound 7, highlighting its therapeutic potential in cancer-associated metabolic dysfunction.

#### 2.3.4 Assessment of hepatic oxidative stress markers

**Fig. 10:**
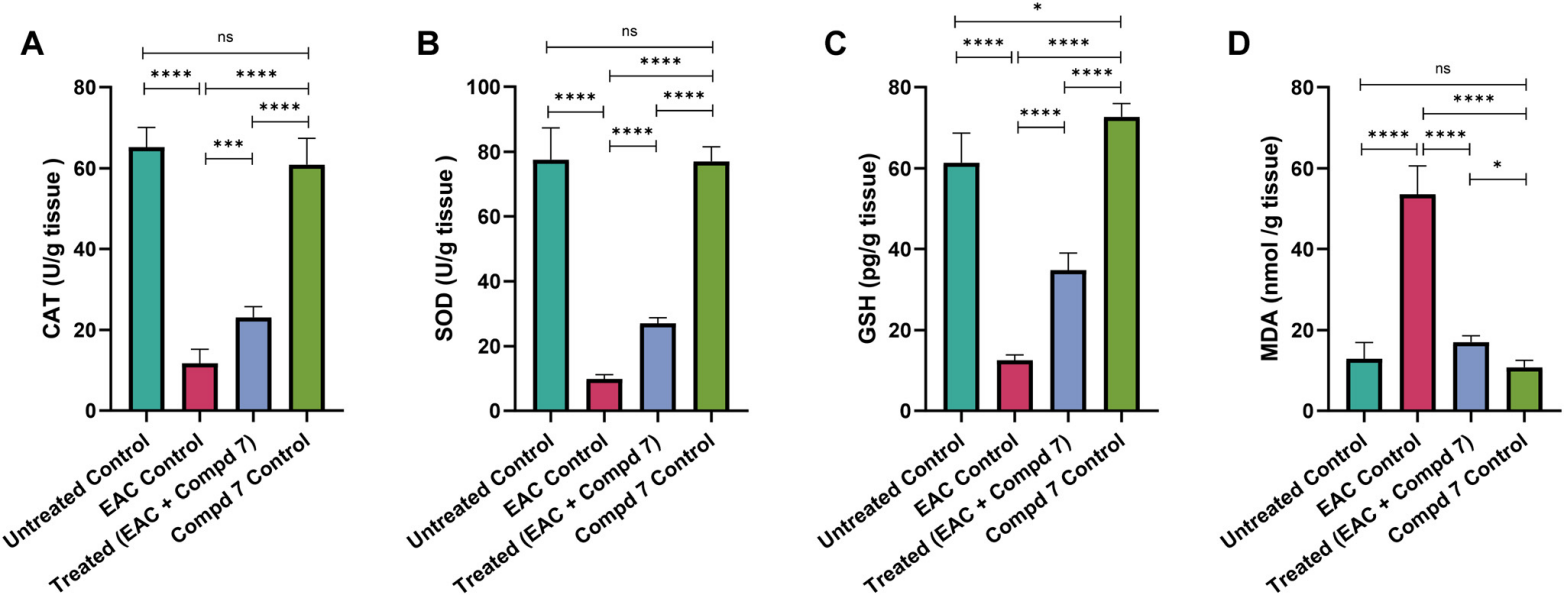
Effect of compound 7 treatment on the expression levels of hepatic oxidative stress ((A) CAT, (B) SOD, (C) GSH and (D) MDA) markers among experimental groups. Data are presented as mean ± SE, with *n* = 6. The statistical analysis was conducted using one-way ANOVA and two-way ANOVA and considered significant at *p* ≤ 0.05 (****p ≤ 0.0001, ***p ≤ 0.001, **p ≤ 0.01, and *p ≤ 0.05).

Oxidative stress is a key contributor to tumor development and tissue damage in cancer models. It results from an imbalance between the generation of reactive oxygen species and the capacity of endogenous antioxidant defenses.^61^ To assess the antioxidant potential of compound 7, we measured levels of reduced glutathione (GSH), catalase (CAT), and superoxide dismutase (SOD) in liver tissue, along with malondialdehyde (MDA), a marker of lipid peroxidation. As shown in Fig. 10, the EAC control group exhibited a significant depletion of antioxidant defenses, with markedly reduced levels of GSH (12.50 ± 0.55 pg g^−1^ tissue), CAT (11.71 ±1.42 U g^−1^ tissue), and SOD (9.85 ± 0.53 U g^−1^ tissue), compared to the untreated control group (GSH: 61.4 ± 2.99; CAT: 65.23 ± 1.97; SOD: 77.56 ± 4.02). In contrast, MDA levels were significantly elevated in the EAC group (53.57 ± 9.6 nmol g^−1^ tissue), indicating extensive lipid peroxidation and oxidative injury (*p* < 0.0001 for all comparisons). Treatment with compound 7 resulted in significant restoration of antioxidant parameters, with increased GSH (34.76 ± 1.71 pg g^−1^ tissue), CAT (23.12 ± 1.09 U g^−1^ tissue), and SOD (27.04 ± 0.70 U g^−1^ tissue) levels, alongside a marked decrease in MDA to 17.04 ± 0.64 nmol g^−1^ tissue (*p* < 0.0001 *vs.* EAC control), indicating effective attenuation of oxidative stress. Notably, the compound 7 control group displayed antioxidant marker levels comparable to or slightly better than the untreated control (GSH: 72.73 ± 1.37; CAT: 60.88 ± 2.66; SOD: 77.05 ± 1.84; MDA: 10.77 ± 0.70), indicating that compound 7 does not induce oxidative stress in healthy liver tissue. Oxidative stress plays a fundamental role in tumor development, immune dysregulation, and tissue injury.^61^ In this context, evaluating the redox balance in liver tissue offers mechanistic insight into the protective efficacy of compound 7. The significant reduction in GSH, CAT, and SOD levels observed in the EAC control group confirms the impairment of the antioxidant defense system in response to tumor-induced oxidative stress. Concurrently, the marked increase in MDA levels reflects intensified lipid peroxidation and membrane damage. These findings are consistent with the known oxidative burden in EAC-bearing animals, where ROS generation contributes not only to cellular damage but also supports tumor progression through genomic instability and chronic inflammation.^62,63^ Treatment with compound 7 reversed these alterations, restoring antioxidant enzyme activity and significantly reducing MDA levels. This antioxidant effect can be attributed to the chemical structure of compound 7, particularly its multiple hydroxyl (–OH) groups, which are known to donate hydrogen atoms and scavenge free radicals. Furthermore, the compound's conjugated π-electron system may enhance resonance stabilization of radical species, limiting oxidative damage and preserving enzymatic function. The presence of bromine atoms could further influence redox activity by modulating the electronic environment and radical quenching properties. The lack of oxidative disruption in the compound 7-control group provides further verification regarding the safety profile of compound 7. Collectively, these findings highlight the role of compound 7 as a potent antioxidant agent capable of mitigating EAC-induced oxidative damage in hepatic tissue.

#### 2.3.5 Assessment of pro-inflammatory TNF-α expression

**Fig. 11:**
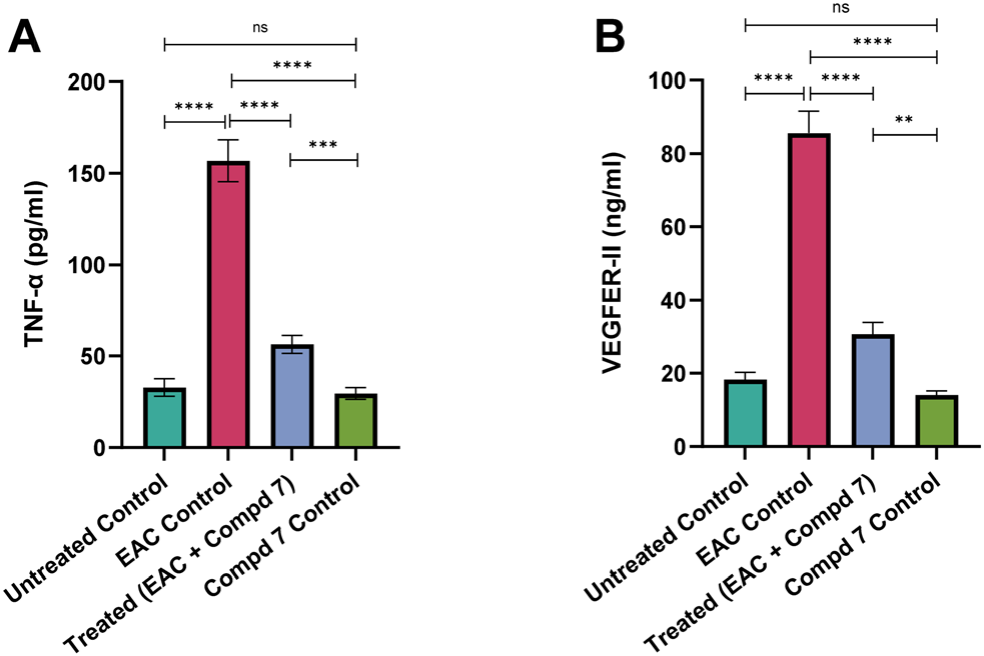
Effect of compound 7 treatment on the expression of serum TNF-α (A) and VEGFR-II (B) levels among different experimental groups. Data are presented as mean ± SE, with *n* = 6. The statistical analysis was conducted using one-way ANOVA and two-way ANOVA and considered significant at *p* ≤ 0.05 (****p ≤ 0.0001, ***p ≤ 0.001, **p ≤ 0.01, and *p ≤ 0.05).

Tumor necrosis factor-alpha (TNF-α) is a pro-inflammatory cytokine that plays a pivotal role in cancer-related inflammation. It promotes tumor cell survival, proliferation, angiogenesis, and immune evasion. Elevated TNF-α levels are typically associated with an aggressive tumor microenvironment and systemic toxicity.^64^ To explore the mechanistic basis of the therapeutic effects of compound 7, we assessed the expression of serum TNF-α levels among the different experimental groups. As illustrated in Fig. 11, serum levels of TNF-α were markedly elevated in the EAC control group, reaching 156.80 ± 4.71 pg ml^−1^, compared to 156.80 ± 4.71 pg ml^−1^ in the untreated control group (*p* < 0.0001). This significant increase reflects the tumor-induced systemic inflammation typically associated with EAC progression. Treatment with compound 7 significantly attenuated TNF-α levels to 56.42 ± 2.01 pg ml^−1^ (*p* < 0.0001 *vs.* EAC control), indicating a strong anti-inflammatory effect. Importantly, the compound 7-only control group exhibited TNF-α levels of 29.37 ± 1.33 pg ml^−1^, statistically comparable to the untreated control group (*p* > 0.05), indicating that compound 7 does not induce inflammatory effects. The elevated TNF-α levels observed in the EAC-bearing mice are consistent with previous findings that link this cytokine to cancer-promoting inflammation. TNF-α contributes to the malignant phenotype by enhancing proliferation, angiogenesis, and resistance to cell death.^65,66^ The significant reduction of TNF-α levels following compound 7 treatment suggests that the compound exerts strong anti-inflammatory effects, which may play a central role in disrupting the tumor-supportive microenvironment. By normalizing TNF-α expression, compound 7 likely reduces immune-mediated tissue damage and tumor aggressiveness. Furthermore, its inability to elevate TNF-α in control animals highlights its non-immunogenic and safe profile.

#### 2.3.6 Assessment of serum angiogenic VEGFR-II expression

Vascular endothelial growth factor-receptor II (VEGFR-II) is a key mediator of angiogenesis and vascular remodeling. Overexpression of VEGFR-II is a hallmark of tumor progression, as it supports neovascularization, tumor nourishment, and metastatic potential. Targeting VEGFR-II is a well-established anti-angiogenic strategy in cancer therapy.^67^ To further explore whether the anticancer potential of compound 7 is associated with its capability to modulate angiogenesis, we assessed the expression of serum VEGFR-II among experimental groups. As shown in Fig. 11, VEGFR-II levels were significantly elevated in the EAC control group (85.61 ± 2.47 ng ml^−1^) compared to the untreated control group (18.23 ± 0.81 ng ml^−1^, *p* < 0.0001), confirming active tumor-driven angiogenesis. Treatment with compound 7 resulted in a marked reduction of VEGFR-II levels to 30.66 ± 1.31 ng ml^−1^ (*p* < 0.0001 *vs.* EAC control), suggesting potent anti-angiogenic activity. The compound 7-only control group showed VEGFR-II levels of 14.03 ± 0.45 ng ml^−1^, which were statistically comparable to the untreated control (*p* > 0.05), confirming that compound 7 does not interfere with physiological angiogenesis in normal tissues. VEGFR-II plays a crucial role in tumor vascularization, enabling rapid growth and metastatic dissemination. The observed upregulation of VEGFR-II in EAC-bearing mice confirms the tumor's angiogenic activity, which is essential for sustaining its progression.^68^ The marked decrease in VEGFR-II expression following treatment with compound 7 supports its anti-angiogenic effect, likely limiting the tumor's access to oxygen and nutrients and thereby impairing its viability. The absence of VEGFR-II suppression in non-EAC-bearing mice suggests that compound 7 selectively targets angiogenesis without disrupting normal vascular homeostasis.

#### 2.3.7 Histological assessments of liver and kidney tissues

**Fig. 12:**
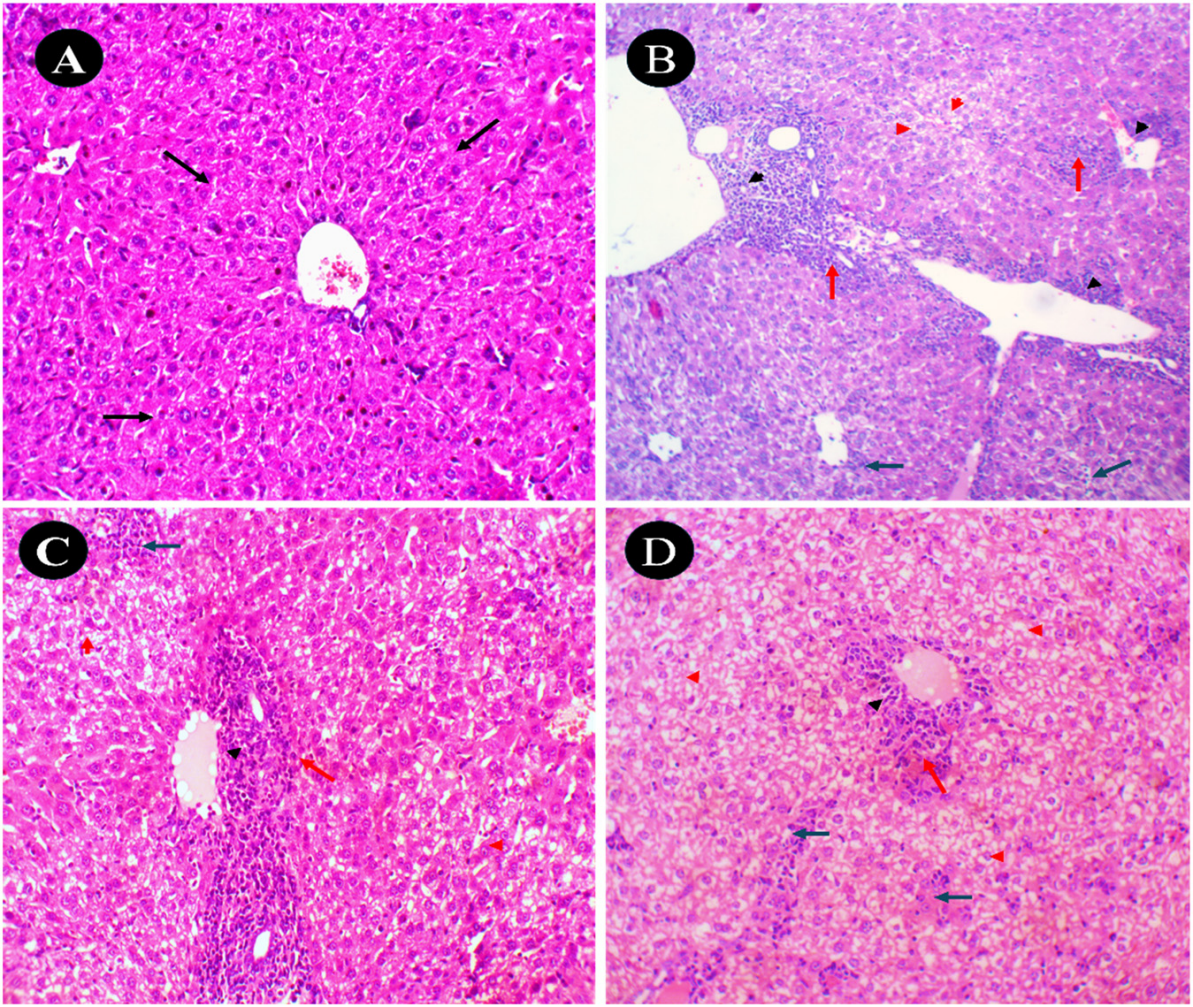
Photomicrographs of liver tissues stained with hematoxylin and eosin (H&E, 20×). (A) Untreated control group: liver tissue exhibits normal architecture with uniform hepatocyte arrangement and no signs of inflammation, degeneration, or fibrosis. (B) EAC control group: displays significant pathological changes, including hydropic degeneration of hepatocytes, lobular inflammation, and marked portal inflammation. (C) Treated group (EAC + compound 7): shows notable histological improvement with reduced inflammatory infiltrates and minimal cellular degeneration, indicating partial hepatoprotection. (D) Compound 7-only control group: demonstrates moderate portal tract expansion with mild inflammatory cell infiltration and preserved overall hepatic structure.

Histological examination of liver tissue stained with hematoxylin and eosin (H&E, 20×) revealed clear differences among the experimental groups, as shown in Fig. 12. Liver slices from the untreated control group displayed a homogenous architecture, with well-preserved and orderly cords of hepatocytes (black arrows), and no evidence of inflammation, cellular damage, or fibrosis, yielding a histopathological score of 0. In contrast, the EAC-treated group exhibited substantial hepatic damage. There was pronounced enlargement of the portal tracts with chronic inflammatory cell infiltrates (black arrowheads), extending into zone 3 hepatocytes (red arrows). Extensive hydropic degeneration of hepatocytes was visible (red arrowheads), accompanied by multiple foci of lobular inflammation (blue arrows). The degeneration affected approximately 45% of the tissue (grade 2), with two lobular inflammatory foci (grade 2) and severe portal inflammation (grade 3). The total liver pathology score was 7, with no signs of fibrosis. In the EAC-induced group treated with compound 7, significant histological improvement was observed. Inflammatory infiltrates within the portal tracts were notably reduced (black arrowheads), and only minimal infiltration extended into zone 3 hepatocytes (red arrows). Hepatocyte hydropic degeneration was limited to around 15% (red arrowheads; grade 1), with no evidence of lobular inflammation and moderate portal inflammation (grade 2). These features indicate a partial but meaningful hepatoprotective effect of compound 7, reflected by a reduced liver pathology score of 3, again without any fibrosis. The control group with compound 7 only also showed largely preserved liver architecture, though with some mild changes. The portal tracts exhibited moderate expansion with minimal infiltration of inflammatory cells (black arrowheads), and scattered hepatocytes showed modest hydropic degeneration (red arrowheads). A single lobular inflammatory focus (blue arrow; grade 1) was noted, and portal inflammation was moderate (grade 2). This group exhibited a liver pathology score of 4, with no fibrosis observed, suggesting that compound 7 has limited intrinsic hepatic toxicity.

**Fig. 13:**
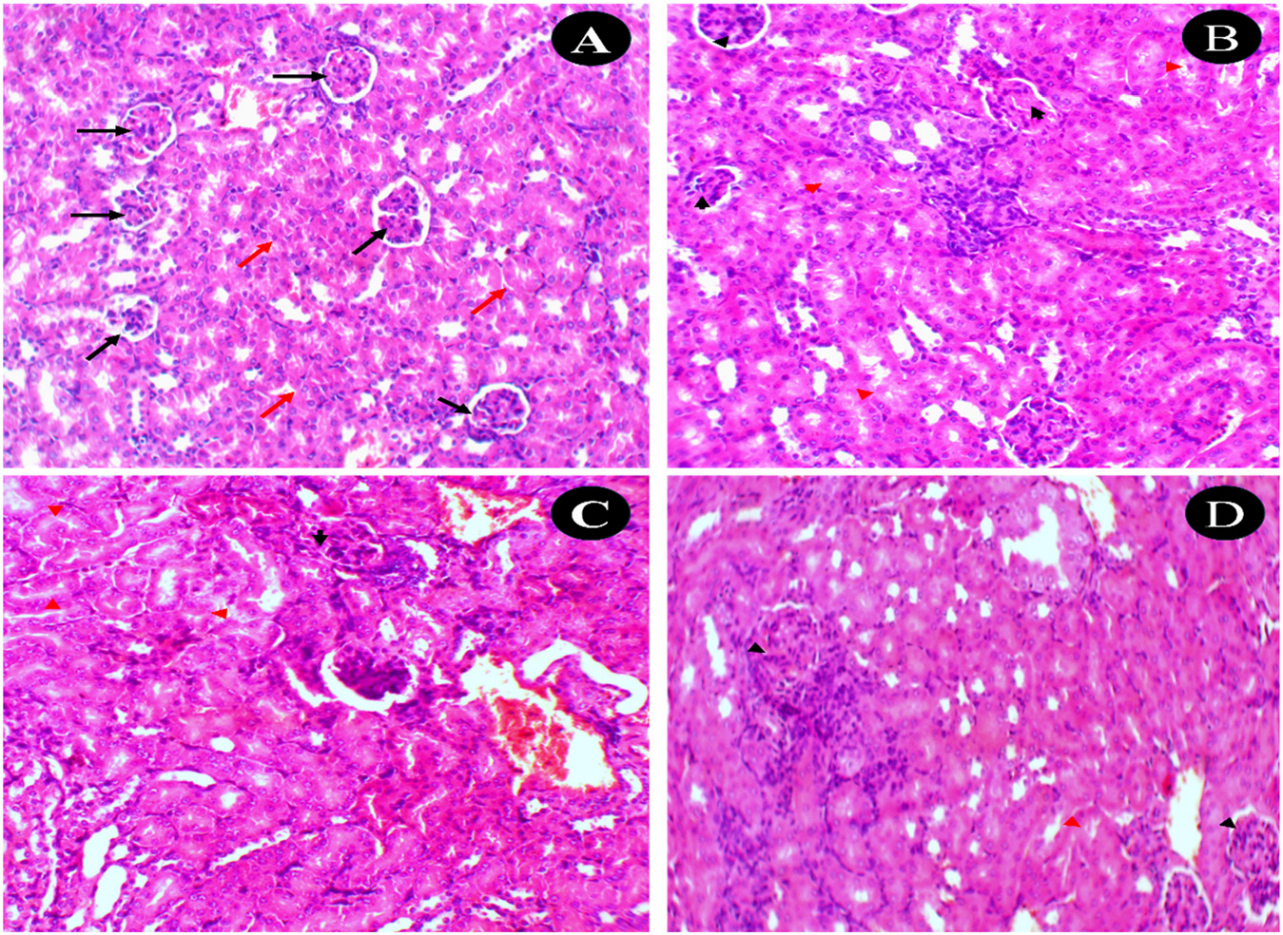
Photomicrographs of kidney tissues stained with hematoxylin and eosin (H&E, 20×). (A) Untreated control group: renal tissue showing normal histology with regular glomeruli and intact tubular architecture, without signs of injury or inflammation. (B) EAC control group: exhibiting pathological alterations including tubular damage and glomerular changes indicative of early nephropathy. (C) Treated group (EAC + compound 7): displaying notable improvement in renal histology, with reduced signs of tubular injury and glomerular damage, suggesting a protective effect. (D) Compound 7-only control group: showing uniform glomeruli and preserved renal structure with no evidence of injury or pathological changes.

Histological analysis of kidney sections (H&E, 20×) showed similar group-dependent differences. In the untreated control group, renal tissue appeared normal, with uniform glomeruli (black arrows) and intact tubular structures (red arrows). There were no signs of injury, inflammation, degeneration, or fibrosis, resulting in a pathological score of 0 (Fig. 13). The EAC-treated group exhibited significant renal pathology. Several glomeruli showed mesangial proliferation with focal sclerosis (black arrowheads), while numerous tubules demonstrated features of acute tubular injury (red arrowheads). The extent of tubular damage was estimated at 30% (grade 2+), and glomerular abnormalities involved approximately 25% of structures, consistent with early nephropathy (class II). In the compound 7 + EAC-treated group, the kidney architecture appeared improved compared to the EAC control. Only a few glomeruli still exhibited mesangial proliferation (black arrowheads), and many tubules retained some features of acute injury (red arrowheads). Glomerular involvement decreased to 15%, still within class II nephropathy criteria, but the degree of injury was clearly attenuated. The control group with compound 7 only displayed generally preserved renal architecture. Occasional glomeruli showed mesangial proliferation with focal sclerosis (black arrowheads), and a few tubules presented mild signs of acute injury (red arrowheads). Overall, the tissue showed no overt pathological signs, suggesting that compound 7 exhibits minimal nephrotoxicity.

These histological evaluations revealed the protective effects of compound 7 against EAC-induced hepatic and renal damage. In the liver, the EAC control group exhibited significant hepatocellular injury, characterized by hydropic degeneration, inflammatory cell infiltration, and portal expansion. These changes are consistent with previous studies indicating that EAC induces liver inflammation and fibrosis.^69,70^ The administration of compound 7 markedly ameliorated these hepatic abnormalities, suggesting its hepatoprotective potential. This protective effect may be attributed to the antioxidative and anti-inflammatory properties of compound 7, which could mitigate oxidative stress and inflammatory responses commonly associated with EAC-induced liver damage.^69–71^ Similarly, in the kidney, the EAC control group displayed pathological features indicative of nephrotoxicity, including mesangial proliferation, glomerular sclerosis, and acute tubular injury. These findings align with research demonstrating that EAC provokes renal toxicity and DNA injury in mice.^59,72,73^ Treatment with compound 7 resulted in significant improvement in renal histology, with reduced glomerular and tubular alterations. This nephroprotective effect may be due to the compound's ability to enhance antioxidant defenses and reduce oxidative damage. Importantly, the control group with compound 7 only exhibited minimal hepatic and renal changes, indicating a favorable safety profile. This observation underscores the compound's low intrinsic toxicity and supports its potential therapeutic application. Collectively, these findings highlight the dual protective and therapeutic potential of compound 7 in mitigating EAC-induced hepatorenal damage.

## 3. Materials and methods

### 3.1 Chemistry

#### 3.1.1 General information

All reagents and solvents were of analytical grade and obtained from Sigma-Aldrich, TIC, or Acros Organics. Deuterated solvents such as DMSO-d_6_ and CDCl_3_ were used for NMR spectroscopy. The ^1^H-NMR (400 MHz) and ^13^C-NMR (100 MHz) spectra were recorded on a Bruker Avance DRX 400 MHz spectrometer. Chemical shifts (*δ*) are given in ppm and referenced to residual solvent peaks. Melting points were measured in open capillary tubes using a Reichert hot-stage apparatus and were uncorrected. IR spectra were acquired utilizing a Shimadzu 470 infrared spectrophotometer with samples prepared as KBr pellets. Elemental analyses were performed on a PerkinElmer 2400 Series I CHN analyzer. Compounds 2, 3, and 4a–c were obtained according to previously reported procedures.^39–41^

#### 3.1.2 Synthetic procedures and characteristics

##### Synthesis of ethyl (*E*)-2-cyano-3-(4-((6,8-dibromo-7-hydroxy-4-methyl-2-oxo-1,2-dihydroquinolin-3-yl)oxy)-3-methoxyphenyl)acrylate (6a)

A solution of compound 5b (1.25 g, 5 mmol) in 50 mL of 2 N sodium hydroxide was heated under reflux at 110 °C for 1 hour. Afterward, compound 3 (2.6 g, 6 mmol) was added, and the reaction mixture was further refluxed for 8 hours, with progress monitored by TLC. Upon completion, the reaction was cooled to 0 °C and quenched by the addition of 1 M hydrochloric acid. The mixture was then left to stand at room temperature for 12 hours. The resulting precipitate was filtered, washed thoroughly with water, and dried under reduced pressure. The crude product was purified by recrystallization from ethanol, yielding compound 6a as pale yellow crystals. Yield: 61%, m.p. 260 °C. IR (KBr) *ν*_max_ (cm^−1^): 3375 (br, OH), 3208 (NH), 1710–1682 (br, C=O), 1608, 1588 (C=C), 1125, 1102, 1046 (C–O). ^1^H-NMR (400 MHz, DMSO-d_6_, ppm) *δ*: 1.28–1.31 (t, 3H, CH_3_), 2.22 (s, 3H, CH_3_), 3.82 (s, 3H, OCH_3_), 4.26–4.31 (q, 2H, CH_2_), 6.86–6.96 (d, 2H, *J* = 8.4 Hz, Ar–H), 7.58–7.75 (d, 2H, *J* = 8.4 Hz, Ar–H), 7.92 (s, 1H, Ar–H), 8.20 (s, 1H, H–olefinic), 10.56 (br. s, 1H, OH), 11.47 (br. s, 1H, NH). ^13^C-NMR (100 MHz, DMSO-d_6_, ppm) *δ*: 157.89, 156.97 (2C=O), 155.47, 154.73, 153.58 (3C–O), 152.78, 151.55, 151.30, 150.42, 150.42, 149.39, 148.25, 143.01, 130.02, 128.63, 127.37, 126.21, 123.26, 122.18 (C–aromatic, C=C, CN), 56.00 (CH_2_), 53.49 (OCH_3_), 19.81 (–CH_3_), 19.73 (–CH_3_). Anal. calcd for C_23_H_18_Br_2_N_2_O_6_ (*M*_wt_ = 578): C, 47.78; H, 3.14; N, 4.84; O, 16.60. Found; C, 47.66; H, 3.02; N, 4.71.

##### Synthesis of ethyl (*E*)-2-cyano-3-(3,5-dibromo-4-((6,8-dibromo-7-hydroxy-4-methyl-2-oxo-1,2-dihydroquinolin-3-yl)oxy)phenyl)acrylate (6b)

Compound 5c (1.5 g, 4 mmol) was dissolved in 40 mL of 2 N sodium hydroxide, and the resulting solution was refluxed at 110 °C for 1 hour. Subsequently, compound 3 (2.1 g, 5 mmol) was added, and the mixture was refluxed for an additional 8 hours, with reaction progress monitored by TLC. Upon completion, the reaction was cooled to 0 °C and quenched by the gradual addition of 1 M hydrochloric acid. The mixture was stirred for 1 hour and then left to stand at room temperature overnight. The solid formed was collected by filtration, thoroughly washed with water, and dried under reduced pressure. Recrystallization from ethanol afforded compound 6b as a yellow solid. Yield: 69%, m.p. 210 °C. IR (KBr) *ν*_max_ (cm^−1^): 3287 (br. OH), 2922, 2826 (C–H), 1728 (br. C=O), 1610, 1590 (C=C), 1222, 1122, 1098, 1043 (C–O). ^1^H-NMR (400 MHz, DMSO-d_6_, ppm) *δ*: 1.28–1.31 (t, 3H, CH_3_), 2.15 (s, 3H, CH_3_), 3.70–3.85 (q, 2H, CH_2_), 6.89 (s, 1H, Ar–H), 7.78 (s, 1H, Ar–H), 7.96 (s, 1H, Ar–H), 8.26 (s, 1H, H–olefinic), 9.88 (s, 1H, NH), 11.60 (br. s, 1H, OH). ^13^C-NMR (100 MHz, DMSO-d_6_, ppm) *δ*: 163.22, 158.80 (2C=O), 157.93, 156.65, 154.21 (3C–O), 152.40, 152.37, 151.67, 150.47, 149.81, 148.63, 143.05, 130.13, 129.63, 129.36, 126.32, 123.78, 122.23, 116.74 (C–aromatic, C=C, CN), 56.74 (CH_2_), 19.96 (–CH_3_), 19.86 (–CH_3_). Anal. calcd for C_22_H_14_Br_4_N_2_O_5_ (*M*_wt_ = 705): C, 37.43; H, 2.00; N, 3.97. Found; C, 37.10; H, 1.91; N, 3.16.

##### Synthesis of ethyl (*E*)-2-cyano-3-(4-((6,8-dibromo-7-hydroxy-4-methyl-2-oxo-1,2-dihydroquinolin-3-yl)oxy)phenyl)acrylate (7)

A solution of compound 5a (3.2 g, 14.8 mmol) in dry ethanol (150 mL) was treated with anhydrous K_2_CO_3_ (6.2 g, 44.2 mmol). After the mixture was allowed to reflux at 85 °C for 1 h, compound 3 (7.6 g, 18.5 mmol) was added in one portion. The obtained reaction was continued refluxing for 12 h under the same conditions (as indicated by TLC analysis). The reaction was subsequently quenched at 0 °C with 1 M HCl and the mixture was stirred for 1 h. The obtained mixture was kept standing at ambient temperature for 16 h, before the precipitate was separated by filtration. The collected solid was washed with water and dried under reduced pressure. Recrystallization of the crude product using ethanol provided the desired compound 7 as faint brown crystals. Yield: 71%, m.p. 250 °C. IR (KBr) *ν*_max_ (cm^−1^): 3400 (br, OH), 3200 (NH), 1720–1680 (br, C=O), 1605, 1585 (C=C), 1130, 1105, 1050 (C–O). ^1^H-NMR (400 MHz, DMSO-d_6_, ppm) *δ*: 1.28–1.31 (t, 3H, CH_3_), 3.83 (s, 3H, CH_3_), 4.26–4.31 (q, 2H, OCH_2_), 6.86–6.98 (d, 2H, *J* = 8.4 Hz, Ar–H), 7.66 (s, 1H, Ar–H), 7.93–7.98 (d, 2H, *J* = 8.00 Hz, Ar–H), 8.20 (s, 1H, H–olefinic), 10.86 (br. s, 1H, OH), 11.49 (br. s, 1H, NH). ^13^C-NMR (100 MHz, DMSO-d_6_, ppm) *δ* 63.42, 157.89 (2C=O), 156.98, 155.42, 154.74 (3C–O), 153.58, 152.48, 151.57, 150.42, 149.40, 134.42, 130.03, 127.75, 126.23, 122.95, 116.92, 113.99, 110.16 (C–aromatic, C=C, CN), 62.43 (CH_2_), 19.75 (–CH_3_), 19.72 (–CH_3_). Anal. calcd for C_22_H_16_Br_2_N_2_O_5_ (*M*_wt_ = 548): C, 48.20; H, 2.94; N, 5.11. Found; C, 48.11; H, 2.88; N, 5.00.

##### Synthesis of ethyl (*E*)-3-(4-((7-acetoxy-6,8-dibromo-4-methyl-2-oxo-1,2-dihydroquinolin-3-yl)oxy)phenyl)-2-cyanoacrylate (8)

A solution of compound 7 (2.2 g, 4 mmol) in 50 mL acetic anhydride was allowed to reflux at 150 °C, and reaction progress was followed up utilizing TLC analysis. After being refluxed for 6 h, the reaction was cooled to 0 °C and subsequently quenched with water. The obtained mixture was stirred for 1 h under the same conditions, and then allowed to stand at ambient temperature overnight. The mixture was filtered, and the collected crude solid was washed with water. After being dried under reduced pressure, the crude solid was recrystallized with ethanol to provide compound 8 as pale yellow crystals. Yield: 76%, m.p. 210 °C. IR (KBr) *ν*_max_ (cm^−1^): 2949 (C–H), 2220 (C≡N), 1718–1684 (br. C=O), 1610, 1519 (C=C, C=N), 1228, 1187, 1094 (C–O), 539, 489 (C–Br). ^1^H-NMR (400 MHz, DMSO-d_6_, ppm) *δ*: 1.21–1.34 (t, 3H, *J* = 7.20 Hz, CH_3_), 1.77 (s, 3H, CH_3_), 1.92 (s, 3H, OCH_3_), 4.26–4.36 (q, 2H, CH_2_), 6.87–7.00 (d, 2H, *J* = 8.8 Hz, Ar–H), 7.36–8.22 (d, 2H, *J* = 8.8 Hz, Ar–H), 8.24 (s, 1H, H–olefinic), 8.41 (s, 1H, NH). ^13^C-NMR (100 MHz, DMSO-d_6_, ppm) *δ*: 172.52, 169.28, 167.41 (3C=O), 163.42, 162.27 (2C–O), 158.90, 156.65, 155.14, 154.46, 152.41, 151.23, 150.47, 134.45, 132.89, 130.09, 129.42, 126.33, 120.30, 116.94, 116.07 (C–aromatic, C=C, CN), 62.89 (CH_2_), 62.43 (OCH_3_), 22.97 (–CH_3_), 14.46 (–CH_3_). Anal. calcd for C_24_H_18_Br_2_N_2_O_6_ (*M*_wt_ = 590): C, 48.84; H, 3.07; N, 4.75. Found; C, 48.70; H, 3.00; N, 4.60.

##### Synthesis of ethyl (*E*)-3-(4-((7-acetoxy-6,8-dibromo-4-methyl-2-oxo-1,2-dihydroquinolin-3-yl)oxy)-3-methoxyphenyl)-2-cyanoacrylate (9)

Compound 6a (1.2 g, 2.1 mmol) was dissolved in 25 mL of acetic anhydride, and the solution was refluxed at 150 °C for 8 hours, with reaction progress monitored by TLC. Upon completion, the reaction was cooled to 0 °C and quenched by the addition of water. The resulting mixture was stirred at this temperature for 1 hour and then left to stand at room temperature overnight. The precipitated solid was collected by filtration, washed thoroughly with water, and dried. The crude product was recrystallized from ethanol to yield compound 9 as yellow crystals. Yield: 63%, m.p. 210 °C. IR (KBr) *ν*_max_ (cm^−1^): 3383 (br. OH), 3215 (NH), 1713–1685 (br. C=O), 1610, 1590 (C=C), 1121, 1098, 1043 (C–O). ^1^H-NMR (400 MHz, DMSO-d_6_, ppm) *δ*: 1.22–1.30 (t, 3H, *J* = 7.20 Hz, CH_3_), 2.20 (s, 3H, CH_3_), 2.31 (s, 3H, CH_3_), 3.38 (s, 3H, OCH_3_), 3.84–3.92 (q, 2H, CH_2_), 7.33–7.35 (d, 1H, *J* = 8.4 Hz, Ar–H), 7.53 (s, 1H, Ar–H), 7.72–7.74 (d, 1H, *J* = 8.00 Hz, Ar–H), 7.85 (s, 1H, Ar–H), 7.85–7.97 (d, 1H, *J* = 8.8 Hz, Ar–H), 8.24 (s, 1H, H–olefinic), 8.41 (s, 1H, NH). ^13^C-NMR (100 MHz, DMSO-d_6_, ppm) *δ*: 169.17, 168.65, 167.40 (3C=O), 162.79, 156.11, 155.56, 154.93 (4C–O), 148.96, 145.38, 143.54, 130.57, 128.94, 127.46, 126.31, 124.31, 122.74, 120.50, 119.53, 110.52, 102.58 (C–aromatic, C=C, CN), 56.45 (CH_2_), 53.87 (–OCH_3_), 21.35 (–CH_3_), 21.01 (–COCH_3_), 20.68 (–CH_3_). Anal. calcd for C_25_H_20_Br_2_N_2_O_7_ (*M*_wt_ = 620): C, 48.41; H, 3.25; N, 4.52. Found; C, 48.21; H, 3.01; N, 4.40.

### 3.2 *In vitro* biological assessments

#### 3.2.1 *In vitro* antiproliferative activity assessment

The cytotoxic effects of the synthesized compounds were evaluated using the MTT assay. MCF-7, MDA-MB 231 (human breast cancer), and MCF-10A (non-tumorigenic breast epithelial) cells were seeded (1–3 × 10^4^ cells per well) and incubated for 24 hours at 37 °C in a humidified atmosphere containing 5% CO_2_.^74^ Following incubation, cells were treated with serial dilutions of the test compounds prepared in DMSO. The final concentration of DMSO in all wells, including controls, was maintained below 0.5% v/v to avoid solvent-related cytotoxicity. Doxorubicin was used as a positive control for comparison of antiproliferative activity. Following 48 hours of treatment, the culture medium was gently removed, and each well received 20 μL of MTT reagent (5 mg mL^−1^ in phosphate-buffered saline). The plates were incubated at 37 °C for 4 hours to facilitate the enzymatic reduction of MTT into formazan by metabolically active cells. After incubation, the solution was discarded, and 100 μL of dimethyl sulfoxide was added. The absorbance of each well was measured at 570 nm using a microplate spectrophotometer. Cell viability was determined by comparing absorbance values to those of the untreated controls and expressed as a percentage. GraphPad Prism software was used to plot dose–response curves and calculate IC_50_ values. Each experiment was performed in triplicate and independently replicated on at least three separate occasions. The results were presented as mean ± SD.^75–77^

#### 3.2.2 Cell cycle assay

Cell cycle progression was examined using a propidium iodide (PI)-based staining protocol, adapted from standard manufacturer instructions and previously described methods. MCF-7 cells were seeded at a density of 2 × 10^5^ cells per well in DMEM and incubated overnight under standard conditions (37 °C, 5% CO_2_). The cells were then treated with compound 7 at its IC_50_ concentration (7.65 μM) for 24 hours. A DMSO-treated group served as the vehicle control. After treatment, cells were collected, washed twice with cold phosphate-buffered saline (PBS), and centrifuged at 2000 rpm for 5 minutes. The pellets were fixed in pre-chilled 70% ethanol and stored at −20 °C for 2 hours. After fixation, cells were washed again with PBS, centrifuged, and resuspended in PI/RNase staining solution. Samples were incubated for 30 minutes at 37 °C in the dark to allow for RNA degradation and proper DNA staining. Flow cytometric analysis was conducted using a BD FACSCalibur™ system (BD Biosciences, USA). PI fluorescence was detected using a 488 nm excitation laser and collected through the FL2 channel (585 nm bandpass filter). Cell cycle phase distribution (G_0_/G_1_, S, and G_2_/M) was analyzed using dedicated software. Each experiment was performed in triplicate and independently replicated on at least three separate occasions. The results were presented as mean ± SD. Statistical comparisons were made using GraphPad Prism, with *p* < 0.05 considered significant.^26^

#### 3.2.3 Annexin V-FITC/PI method

Apoptotic cell death was quantified using annexin V-FITC and propidium iodide (PI) staining, in accordance with the manufacturer's instructions (BioVision, USA; Cat No. K101-25), with minor protocol adjustments. MCF-7 cells were plated at a density of 2 × 10^5^ cells per well in DMEM-containing supplements and incubated overnight at 37 °C in a humidified atmosphere with 5% CO_2_. The following day, cells were exposed to compound 7 at its IC_50_ concentration (7.65 μM) for 24 hours. After treatment, cells were harvested using 0.25% trypsin–EDTA, rinsed twice with ice-cold phosphate-buffered saline (PBS), and resuspended in 500 μL of annexin V binding buffer. To each sample, 5 μL of annexin V-FITC and 5 μL of propidium iodide (PI) were then added for staining. The staining mixture was incubated in the dark for 15 minutes at room temperature. Samples were analyzed on a BD FACSCalibur™ flow cytometer (BD Biosciences, USA). Fluorescence was detected using the FL1 channel (530 nm) for annexin V-FITC and the FL2 channel (585 nm) for PI. Quadrant gating was used to distinguish between viable cells (annexin V^−^/PI^−^), early apoptotic cells (annexin V^+^/PI^−^), late apoptotic cells (annexin V^+^/PI^+^), and necrotic cells (annexin V^−^/PI^+^). Each experiment was performed in triplicate and independently replicated on at least three separate occasions. The results were presented as mean ± SD with statistical significance evaluated using GraphPad Prism software.^78^

#### 3.2.4 Gene expression analysis by qRT-PCR

Total RNA was extracted from MCF-7 cells following 24 hour treatment with compound 7 at its IC_50_ concentration (7.65 μM) using the RNeasy Mini Kit (Qiagen, Valencia, CA, USA), according to the manufacturer's instructions. Untreated cells served as negative controls. RNA concentration and purity were verified spectrophotometrically prior to cDNA synthesis. Complementary DNA (cDNA) was synthesized from purified RNA using the RT^2^ First Strand Kit (Qiagen, Valencia, CA, USA) following the supplied protocol. Quantitative real-time PCR (qRT-PCR) was performed on a Corbett Rotor-Gene 6000 system (Qiagen, USA) using the RT^2^ SYBR® Green ROX™ FAST Master Mix (Qiagen). Each 25 μL reaction contained 12.5 μL of master mix, 1 μL of gene-specific primers (RT^2^ qPCR Primer Assays, Qiagen), 1 μL of cDNA template, and 10.5 μL of RNase-free water. Gene-specific primers were selected using the PrimerBank database for BAX, BCL2, and TP53, along with GAPDH as the endogenous control (Table S3). Thermal cycling conditions were set as follows: initial enzyme activation at 95 °C for 10 minutes, followed by 40 cycles of denaturation at 95 °C for 15 seconds, and annealing at 60 °C for 30 seconds. Amplification specificity was confirmed by melting curve analysis. Gene expression levels were normalized to GAPDH mRNA as the internal reference, and relative quantification was performed using the ΔΔCt method. Each experiment was performed in triplicate and independently replicated on at least three separate occasions. The results were presented as mean ± SD. Statistical analysis was performed using GraphPad Prism software, with *p* < 0.05 considered statistically significant.^79^

#### 3.2.5 Tubulin polymerization assay

The effect of compound 7 on tubulin polymerization was evaluated using a standardized fluorescence-based assay in 96-well microplates, following established protocols with minor modifications.^80,81^ Compound 7 and the reference inhibitor, combretastatin A-4 (CA-4), were appropriately diluted in assay buffer and tested in triplicate. A solution of purified tubulin (Cytoskeleton Inc., Cat. no: T240-DX) was freshly prepared at a concentration of 1 mg per 100 μL. The polymerization reaction was initiated by adding a polymerization buffer composed of 80 mM PIPES (pH 6.9), 1 mM MgCl_2_, 1 mM EGTA, and 1 mM GTP, achieving a final reaction volume of 100 μL in each well. Plates were incubated at 37 °C, and tubulin polymerization was monitored in real-time using a microplate reader. Fluorescence readings were recorded every minute for 60 minutes at 360 nm excitation and 420 nm emission wavelengths. Blank wells containing buffer without inhibitors were used as negative controls. The resulting fluorescence intensity values were analyzed using GraphPad Prism, and the extent of tubulin polymerization inhibition was calculated relative to the control. All experiments were conducted in biological triplicates, and data are presented as mean ± SD.

#### 3.2.6 Western blot analysis

Western blot analysis was conducted to evaluate the expression levels of α-tubulin (55 kDa) and β-actin (42 kDa, loading control) in MCF-7 breast cancer cells treated with compound 7 compared to untreated controls. Cells were lysed in RIPA buffer (Thermo Scientific, Cat. #89900) containing protease and phosphatase inhibitors, and lysates were centrifuged at 12 000 × *g* for 15 min at 4 °C. Protein concentrations were determined using the BCA assay (Thermo Scientific, Cat. #23225), and equal amounts (20–50 μg) were mixed with 2× Laemmli sample buffer (Bio-Rad, Cat. #1610737) and denatured at 95 °C for 5 min. Proteins were separated on SDS-PAGE gels (8–10% for α-tubulin, 12% for β-actin; Bio-Rad TGX™, Cat. #456-9033) and transferred to PVDF membranes (Immobilon-P, Millipore, Cat. #IPVH00010) using a wet transfer system (100 V, 1 h, 4 °C). Membranes were blocked with 5% non-fat milk in TBST for 1 h at room temperature and incubated overnight at 4 °C with anti-α-tubulin (1 : 1000, Cell Signaling Technology, Cat. #3873) and anti-β-actin (1 : 5000, Sigma-Aldrich, Cat. #A5441) antibodies. After washing, membranes were incubated with HRP-conjugated secondary antibodies (15 000–1 : 10 000, Cell Signaling Technology) for 1 h at room temperature. Protein bands were visualized using the SuperSignal™ West Pico/Femto ECL substrate (Thermo Scientific, Cat. #34095) and imaged with a Bio-Rad ChemiDoc system. Band intensities were quantified by densitometry using ImageJ software and normalized to β-actin levels.^82–84^

#### 3.2.7 FRAP (ferric reducing antioxidant power) assay

The antioxidant potential of compound 7 was evaluated using the FRAP (ferric reducing antioxidant power) assay kit (MAK369, Sigma-Aldrich), based on the reduction of ferric ions (Fe^3+^) to ferrous ions (Fe^2+^) by antioxidants under acidic conditions. The resulting Fe^2+^ forms a colored complex with the FRAP chromogenic probe, which can be quantitatively measured. The reaction mixture was prepared by combining FRAP assay buffer, FRAP probe, and FeCl_3_ solution as per the manufacturer's protocol. A volume of 50 μL of compound 7 or ascorbic acid (at different concentrations) was transferred into the wells of a 96-well plate, followed by the addition of 150 μL of the prepared reaction mix. Absorbance was measured at 594 nm using a microplate reader after incubation at 37 °C for 60 minutes. Antioxidant activity was determined by comparing the absorbance of the samples to a standard curve generated using known concentrations of the ferrous standard provided in the kit. Each experiment was performed in triplicate and independently replicated on at least three separate occasions. The results were presented as mean ± SD.^85^

#### 3.2.8 Assessment of COX-1 and COX-2 activity

The inhibitory activity of compound 7 against COX-1 and COX-2 was evaluated using BioVision's COX-1 (Cat. #K548-100) and COX-2 (Cat. #K547-100) Inhibitor Screening Kits, which quantify prostaglandin G_2_ production from arachidonic acid *via* a fluorometric assay (Ex/Em = 535/587 nm). Briefly, compound 7, dissolved in DMSO and diluted to a working concentration in COX assay buffer, was added (10 μL) to each well of a 96-well plate. Enzyme control wells received 10 μL of assay buffer, while inhibitor control wells were prepared using 2 μL of celecoxib (reference inhibitor) and 8 μL of assay buffer. COX-1 and COX-2 enzymes were reconstituted in sterile water (110 μL) and kept on ice until use. Arachidonic acid was activated by mixing 5 μL with 5 μL of NaOH, followed by a 10-fold dilution with water and vortexing. A reaction mixture (80 μL per well) was prepared by combining 76 μL of assay buffer, 1 μL of COX probe, 2 μL of 200-fold diluted COX cofactor, and 1 μL of COX-1 or COX-2 enzyme. This mix was added to each well, followed by 10 μL of the prepared arachidonic acid/NaOH solution to initiate the reaction. Fluorescence was recorded at 25 °C using a microplate reader. The percent inhibition was determined by comparing the fluorescence increase in treated wells relative to the enzyme control. IC_50_ values were calculated from the concentration–response curves using GraphPad Prism.

### 3.3 *In silico* molecular modelling analysis

The crystal structures of targeted proteins including tubulin, COX-1, and COX-2 proteins were selected and downloaded from the protein data bank (PDB: *6pc4*, *1eqg*, and *4ph9*, respectively).^86–88^ The 2D and 3D structures of compound 7 were acquired from the Chemdraw program and Discovery Studio 2021, respectively. The structure of compound 7 was subjected to a process of energy minimization followed by geometrical verification. The crystal structures of targeted proteins were also subjected to a preparation process, including removing non-essential components, including extra chains and water molecules, and adding hydrogen atoms to mimic physiological conditions. This was performed in line with standard preparation workflows reported in earlier studies.^89–92^ The docking simulations utilized the triangle matcher algorithm for ligand placement, with rigid receptor refinement applied afterward. London dG and GBVI/WSA dG scoring functions were used to evaluate and rank binding interactions based on predicted affinity. The docking protocol was validated by re-docking the native ligands into their original binding sites. The accuracy of the protocol was confirmed by comparing the predicted poses with experimentally resolved conformations from the crystal structures. Following validation, the same docking workflow was applied to assess the binding interaction of compound 7 with the active sites of the selected proteins, aiming to explore its multi-target inhibitory potential.

### 3.4 Animal-model and molecular assessments

#### 3.4.1 Experimental animals

Adult female Swiss albino mice (20–25 g) were obtained from the Abo Rawash Animal Breeding Facility (Giza, Egypt) for use in this study. Upon arrival, the mice were accommodated in stainless steel mesh cages within the Animal House Facility at the Faculty of Science, Port Said University. They were kept under standard laboratory conditions, including a temperature of 22 ± 2 °C and a 12 hour light/dark cycle, with unrestricted access to food (standard pellet diet) and water. A seven-day acclimatization period was provided prior to the initiation of experimental procedures. All animal handling and experimental protocols were approved by the Animal Ethical Committee of the Faculty of Science, Port Said University, Egypt (Approval No. ERN:PSU.Sci.21), and were conducted in accordance with institutional and national guidelines for the care and use of laboratory animals.

#### 3.4.2 Assessment of EAC cell viability, count, and volume

Ehrlich ascites carcinoma (EAC) cells were initially obtained from the National Cancer Institute (Cairo, Egypt). For routine propagation and maintenance, EAC cells were preserved as ascitic fluid in the peritoneal cavity of female Swiss albino mice *via* serial intraperitoneal (i.p.) passages at intervals of 8–10 days. To evaluate tumor burden in the experimental groups, ascitic fluid was collected from the peritoneal cavity using a sterile, graduated syringe. The volume of ascitic fluid was recorded for each animal. The collected fluid was then diluted with heparinized saline for viable cell counting. Cell viability and density were determined using the trypan blue exclusion method. A 0.4% (w/v) trypan blue solution was prepared by dissolving 0.4 g of dye in 100 mL of sterile distilled water and filtering it through a 0.22 μm membrane filter. Prior to counting, 10 μL of the ascitic cell suspension was mixed with an equal volume of trypan blue and incubated at room temperature for 2 minutes. Cells were loaded onto a hemocytometer (Thoma type, Germany) and examined under a light microscope at 40× magnification. Non-viable cells stained blue, whereas viable cells remained unstained. All measurements were performed in triplicate, and the results are expressed as mean ± SD.^93^

#### 3.4.3 Acute toxicity and lethal dose determination

The acute toxicity of compound 7 was evaluated in Swiss albino mice using the method described by Meier and Theakston, with minor modifications.^94^ Mice were randomly divided into dose groups (*n* = 4 per group), with compound 7 administered in increasing concentrations ranging from 1 to 200 mg kg^−1^ in a final injection volume of 150 μL per mouse. In the first phase, animals were injected with low doses (1, 1.5, 2, 2.5, 3, 3.5, 4, 6, 8, and 10 mg kg^−1^) and observed for 24 hours. Since no mortality was observed, a second set of animals was treated with higher doses (15, 25, 50, 75, 100, and 200 mg kg^−1^), with continuous monitoring for 48 hours post-injection. Control animals received sterile saline solution only. All mice were carefully observed for signs of toxicity, behavioral changes (*e.g.*, grooming, feeding, and locomotion), and mortality. The maximum tolerated dose (LD_0_) was defined as the highest dose at which no mortality or significant toxic symptoms were observed, while the lethal dose (LD_100_) was identified as the dose at which all animals succumbed. These values were used to establish safe dosing limits for subsequent *in vivo* pharmacological and biochemical experiments.

#### 3.4.4 Assessment of effective *in vivo* dose

The effective *in vivo* dose of compound 7 was determined based on its ability to reduce the number of viable EAC cells in a mouse model. Female Swiss albino mice (20–25 g) were randomly assigned to six groups (*n* = 6 per group). On day 1, all groups were injected i.p. with 2.5 × 10^6^ viable EAC cells in 200 μL of sterile saline. Group 1 (EAC control) received EAC cells only and no further treatment. Groups 2–6 received compound 7 at increasing doses of 2.5, 5, 7.5, 10, and 15 mg kg^−1^, respectively. The compound was administered i.p. every other day (*i.e.*, on days 2, 4, 6, 8, and 10), for a total of five doses over a 10 day treatment period. At the end of the experiment (day 11), ascitic fluid was collected from the peritoneal cavity of each animal. The number and viability of EAC cells were assessed using the trypan blue exclusion assay. Each experiment was performed in triplicate and independently replicated on at least three separate occasions. The results were presented as mean ± SD. Statistical comparisons between groups were conducted using GraphPad Prism, with *p* < 0.05 considered significant.^95^

#### 3.4.5 Experimental design, treatment, and tissue collection

Following dose optimization and acute toxicity testing, the *in vivo* anticancer efficacy of compound 7 was investigated using an EAC mouse model. A total of 24 female Swiss albino mice (20–25 g) were randomly divided into four experimental groups (*n* = 6 per group), as follows:

■ Group 1 (untreated control): mice received i.p. injections of sterile saline (0.9% NaCl) every other day for 10 days.

■ Group 2 (compound 7 control): mice were administered compound 7 (10 mg kg^−1^, i.p.) every other day for a total of five doses over 10 days without EAC induction.

■ Group 3 (EAC control): mice were injected i.p. with 2.5 × 10^6^ viable EAC cells in 200 μL of sterile saline on day 1 and received no additional treatment.

■ Group 4 (EAC + compound 7 treated): mice were injected with 2.5 × 10^6^ EAC cells on day 1, followed by compound 7 (10 mg kg^−1^, i.p.) starting on day 2 and continuing every other day for a total of five doses over 10 days.

All animals were maintained under conventional laboratory conditions, including a controlled temperature of 22 ± 2 °C and a 12 hour light/dark photoperiod, with continuous access to standard pellet chow and water. On day 11, following the final treatment, ascitic fluid was collected from the peritoneal cavity using heparinized syringes for EAC cell viability and count analysis. Blood samples were collected under light ether anesthesia *via* the retro-orbital venous plexus and centrifuged at 3000 rpm for 10 minutes. The resulting serum was separated and stored at −20 °C for biochemical analysis. Additionally, liver and kidney tissues were excised from each animal for further evaluation. Each organ was divided into two parts: one portion was rinsed with ice-cold phosphate-buffered saline (PBS, pH 7.4) and stored at −20 °C for preparation of tissue homogenates used in biochemical assays. The second portion was fixed in 10% neutral buffered formalin for histopathological examination to assess tissue morphology and damage.^96,97^

#### 3.4.6 Assessment of liver function enzymes (ALT and AST)

Serum alanine aminotransferase (ALT) and aspartate aminotransferase (AST) levels were measured as indicators of liver function using commercially available assay kits (BioVenic, NY, USA, (AST) Cat. No.: IVEK3681, (ALT) Cat. No.: IVEK3446), in accordance with the manufacturer's instructions. In the ALT assay, the enzyme catalyzes the conversion of alanine to pyruvate, which is then reduced to lactate by lactate dehydrogenase, resulting in the oxidation of NADH to NAD^+^. This reaction causes a decrease in absorbance at 340 nm, which is proportional to ALT activity. Similarly, the AST assay is based on the transamination of aspartate to oxaloacetate, which then reacts with NADH in the presence of malate dehydrogenase, leading to a decrease in NADH absorbance at 340 nm. For each assay, 10 μL of serum was added to the respective substrate buffer and incubated at 37 °C for 30 minutes. The change in absorbance was recorded using a microplate reader at 340 nm. Enzymatic activities were calculated and expressed in units per liter (U L^−1^). All measurements were performed in triplicate, and results are presented as mean ± SD.^98,99^

#### 3.4.7 Evaluation of renal function markers (creatinine and urea)

Serum creatinine and urea levels were assessed as indicators of renal function using colorimetric assay kits from BioAssay Systems (DICT-500, USA), according to the manufacturers' protocols. Creatinine concentration was determined based on a colorimetric enzymatic reaction. In this assay, creatinine is enzymatically converted to creatine and then to sarcosine, which is oxidized to produce hydrogen peroxide. The resulting hydrogen peroxide reacts with a chromogenic substrate in the presence of peroxidase to generate a colored product, the intensity of which is proportional to the creatinine concentration. Briefly, 10 μL of serum was mixed with 200 μL of working reagent, incubated at 25 °C for 5 minutes, and the absorbance was recorded at 510 nm using a microplate reader. Values were quantified against a standard curve and expressed in mg dL^−1^. On the other hand, urea levels were measured based on an enzymatic reaction that produces a chromophore detectable at 340 nm. Specifically, 5 μL of serum was added to 200 μL of the assay reagent and incubated at 37 °C for 10 minutes. Absorbance was measured at 340 nm, and urea concentration was calculated using a standard curve and expressed as mg dL^−1^. All samples were analyzed in triplicate, and data are presented as mean ± SD.^100,101^

#### 3.4.8 Assessment of oxidant and antioxidants markers

##### 3.4.8.1 Catalase (CAT) activity assay

CAT activity was quantified in liver tissue homogenates using a commercial ELISA kit (MyBioSource, USA; Cat. No. MBS006963), following the manufacturer's instructions.^102^ Liver tissues were homogenized in an appropriate lysis buffer and centrifuged to obtain the supernatant. Samples were loaded into ELISA plates pre-coated with catalase-specific antibodies and incubated at 37 °C. After washing steps, a biotinylated detection antibody and horseradish peroxidase (HRP)-conjugated secondary antibody were sequentially added. The reaction was developed using a tetramethylbenzidine (TMB) substrate and stopped with sulfuric acid. Absorbance was measured at 450 nm using a microplate reader, and results were expressed as units per gram of tissue (U g^−1^ tissue). All samples were run in triplicate, and data were reported as mean ± SD.

##### 3.4.8.2 Malondialdehyde (MDA) assay

MDA was measured in liver tissue homogenates using a commercial ELISA kit (MyBioSource, USA; Cat. No. MBS268427), following the manufacturer's instructions.^103^ The assay employed a double-antibody sandwich ELISA technique, where wells are pre-coated with MDA-specific monoclonal antibodies. Liver tissue homogenate samples were added to the wells and incubated to allow specific antigen–antibody binding. After washing, a biotin-labeled polyclonal detection antibody was added, followed by an avidin–HRP conjugate. The enzymatic reaction was developed using a TMB substrate, yielding a colorimetric change proportional to the MDA concentration. The reaction was stopped with acid, and absorbance was measured at 450 nm using a microplate reader. Results were expressed as nanomole per gram of tissue (nmol g^−1^ tissue). All measurements were performed in triplicate, and results are presented as mean ± SD.

##### 3.4.8.3 Glutathione (GSH) assay

GSH was quantified in liver tissue homogenates using a commercial ELISA kit (Shanghai BlueGene Biotech Co., Ltd., China; Cat. No. E02G0367), following the manufacturer's instructions.^104^ The assay employs a sandwich ELISA technique in which GSH in the samples binds to monoclonal antibodies pre-coated on microplate wells. Following this, an HRP-conjugated detection antibody was added, and the plate was incubated at 37 °C for 1 hour. After several washing steps to remove unbound reagents, a TMB substrate solution was added, allowing the enzymatic colorimetric reaction to develop. The reaction was stopped with an acidic solution, and absorbance was measured at 450 nm using a microplate reader. The resulting color intensity was directly proportional to the GSH concentration in the sample, and results were expressed as picogram per gram of tissue (pg g^−1^ tissue). All samples were assayed in triplicate, and results are reported as mean ± SD.

##### 3.4.8.4 Superoxide dismutase activity (SOD) assay

Hepatic SOD activity was assessed using a commercial ELISA kit (MyBioSource, USA; Cat. No. MBS036924), following the manufacturer's guidelines.^105^ The assay employed a sandwich ELISA format, in which SOD present in the tissue homogenates binds to a specific monoclonal antibody pre-coated on microplate wells. After sample incubation at 37 °C for 60 minutes, an HRP-conjugated secondary antibody was added. Following thorough washing, the enzymatic reaction was initiated with a TMB substrate and stopped with an acidic solution, resulting in a measurable color change. Absorbance was recorded at 450 nm using a microplate reader, and results were expressed as units per gram of tissue (U g^−1^ tissue). All samples were run in triplicate, and data were reported as mean ± SD.

#### 3.4.9 TNF-α quantification

Serum levels of TNF-α were quantified using a commercial mouse TNF-α ELISA kit (Elabscience®, China; Cat. No. E-EL-M3063), based on the sandwich ELISA principle, according to the manufacturer's instructions.^106^ Briefly, 96-well microplates pre-coated with a TNF-α-specific capture antibody were loaded with standards and serum samples, followed by incubation to allow antigen binding. After washing, a biotin-labeled detection antibody was added, followed by an avidin–HRP conjugate to amplify the signal. The enzymatic reaction was developed using a TMB substrate and stopped with an acidic solution. Absorbance was measured at 450 nm using the ELISA microplate reader. TNF-α concentrations were calculated by plotting sample absorbance values against a standard curve and expressed in pg mL^−1^. All measurements were performed in triplicate, and data were presented as mean ± SD.

#### 3.4.10 VEGFR-2 levels quantification

Serum VEGFR-2 levels were quantified using a rat VEGFR-2 ELISA kit (Cusabio®, China; Catalog No. CSB-E07348r), based on a quantitative sandwich enzyme immunoassay format. Samples were added to 96-well plates pre-coated with a monoclonal antibody specific for VEGFR-2 and incubated to enable antigen binding. After washing to remove unbound material, a biotin-conjugated detection antibody was added, followed by an avidin–HRP complex. The enzymatic reaction was developed using TMB substrate and stopped with an acidic solution. Absorbance was measured at 450 nm using a microplate reader. The VEGFR-2 concentrations in the samples were calculated using a standard curve fitted with a four-parameter logistic (4-PL) regression model and expressed in nanograms per milliliter (ng mL^−1^). All measurements were performed in triplicate, and results are presented as mean ± SD.^107^

#### 3.4.11 Histopathological examination

Liver and kidney tissues were collected from all experimental groups at the end of the study and immediately fixed in 10% neutral buffered formalin. After fixation, the samples were dehydrated, embedded in paraffin, and sectioned at a thickness of 5 μm using a rotary microtome. The tissue sections were mounted on glass slides and stained with hematoxylin and eosin (H&E) for routine microscopic evaluation.^90,108,109^ Histopathological examination of renal tissue focused on identifying acute tubular injury (ATI), characterized by tubular epithelial cell flattening, necrosis, apical vacuolization, and loss of the brush border. These features were assessed using a semi-quantitative scoring system.^110^ Hepatic tissue sections were evaluated for the severity of hepatocellular injury using a modified Brunt grading system, which includes assessment of hepatocyte ballooning, necrosis, inflammation, and other degenerative changes. Microscopic analysis was performed using a Nikon Eclipse E800 microscope equipped with a digital camera for documentation.^111,112^

#### 3.4.12 Statistical analysis

GraphPad Prism (version 10.4.1, GraphPad Software, USA) was utilized for all statistical analyses. Before analysis, the Shapiro–Wilk test was applied to validate the data for normality. Tukey's *post hoc* test for multiple comparisons was employed following one-way analysis of variance (ANOVA) for comparisons involving more than two groups. When appropriate, two-way ANOVA was used to evaluate interactions between two independent variables. The following criteria were used to determine whether a *p* <0.05 was statistically significant: *p ≤ 0.05, **p ≤ 0.01, ***p ≤ 0.001, ****p ≤ 0.0001. Every experiment was performed in triplicate, and the mean ± SD was employed to express the results.

### 3.5 Institutional review board statement

All experimental procedures were conducted in accordance with the guidelines of the Institutional Animal Care and Use Committee (IACUC) of Port Said University, Egypt. The study protocol was reviewed and approved by the Research Ethics Committee, Faculty of Science, Port Said University (Approval No: ERN:PSU.Sci.21). Efforts were made to minimize animal suffering and to reduce the number of animals used.

## 4. Conclusion

In conclusion, this study introduces a novel class of 7-hydroxy azacoumarin–α-cyanocinnamate hybrids, synthesized through a rational design strategy aimed at integrating key pharmacophores with proven anticancer relevance. Comprehensive structural characterization confirmed the successful formation of the envisioned hybrids. Among the synthesized compounds, compound 7 emerged as the most promising candidate, exhibiting potent and selective cytotoxicity against MCF-7 breast cancer cells, significantly surpassing the reference drug doxorubicin in both efficacy and selectivity index. Mechanistic *in vitro* studies revealed that compound 7 exerts its antiproliferative effect through a dual mechanism involving G_2_/M cell cycle arrest and induction of mitochondrial-mediated apoptosis, as evidenced by upregulation of p53 and Bax, along with suppression of the anti-apoptotic Bcl-2 gene. Furthermore, compound 7 significantly inhibited tubulin polymerization, suggesting interference with microtubule dynamics as a potential mode of action. The hybrid scaffold also conferred antioxidant capacity, demonstrated by dose-dependent FRAP activity, and displayed selective inhibition of the COX-2 enzyme, supporting its potential to modulate oxidative stress and inflammation, two critical hallmarks of tumor progression. These multi-targeted effects were corroborated by *in vivo* validation using the EAC mouse model, where compound 7 significantly reduced tumor cell viability and ascitic tumor volume in a dose-dependent manner. Notably, compound 7 provided substantial protection against EAC-induced hepatorenal dysfunction, as demonstrated by restored liver/kidney biomarkers, improved oxidative stress profiles (GSH, CAT, SOD, MDA), and suppressed systemic inflammation and angiogenesis markers (TNF-α, VEGFR-II). Histological evaluations further confirmed the compound's protective effect on liver and kidney architecture, with minimal intrinsic toxicity in healthy animals. Altogether, these findings provide compelling evidence that compound 7 represents a highly promising lead candidate with multi-faceted anticancer potential. Its ability to simultaneously target cell cycle progression, apoptotic signaling, oxidative stress, inflammation, and angiogenesis underscores its value as a lead structure for the development of potent multi-target anticancer agents.

## Author contributions

Conceptualization: M. A. E., I. M. E., and E. M. S.; methodology: M. A. E., I. M. E., R. M. M., T. A. Y., A. A. A., and E. M. S.; software: M. A. E., R. M. M., T. A. Y., and E. M. S.; validation: M. A. E., I. M. E., R. M. M., T. A. Y., A. A. A., and E. M. S.; formal analysis: M. A. E., I. M. E., R. M. M., T. A. Y., and E. M. S.; investigation: M. A. E., I. M. E., and E. M. S.; resources: M. A. E., I. M. E., R. M. M., and E. M. S.; data curation: M. A. E., R. M. M., T. A. Y., A. A. A., and E. M. S.; writing – original draft preparation: M. A. E., I. M. E., R. M. M., T. A. Y., A. A. A., and E. M. S.; writing – review and editing: M. A. E., I. M. E., R. M. M., T. A. Y., A. A. A., and E. M. S.; visualization: M. A. E., R. M. M., A. A. A., and E. M. S.; supervision: M. A. E., I. M. E., and E. M. S.; project administration: M. A. E., I. M. E., R. M. M., and E. M. S.; funding acquisition: M. A. E., I. M. E., R. M. M., A. A. A., and E. M. S. All authors have read and agreed to the published version of the manuscript.

## Conflicts of interest

The authors affirm that they are not aware of any personal or financial conflicts that would have seemed to affect the findings of this study's research.

## Supplementary Material

MD-016-D5MD00484E-s001

MD-016-D5MD00484E-s002

MD-016-D5MD00484E-s003

## Data Availability

Table S1: summary of IC_50_ values for the antiproliferative activity of the synthesized compounds (6a, 6b, 7, 8, and 9) and doxorubicin toward MCF-7, MDA-MB 231, and MCF-10A cells. Table S2: the binding affinity scores and interactions of compound 7 and co-crystallized ligands toward tubulin, COX-1, and COX-2 proteins. Table S3: the list of primers utilized in gene expression assessment using RT-PCR. Fig. S1: *in vitro* antiproliferative activity of compound 7 and doxorubicin toward MCF-10A cells. Fig. S2: effect of compound 7 (at its IC50 value) on the expression of tubulin protein in MCF-7 cells. Fig. S3–S7: ^1^H-NMR and ^13^C-NMR spectrum of compounds 6a, 6b, 7, 8, and 9, respectively. See DOI: https://doi.org/10.1039/D5MD00484E. Data supporting the results reported in this manuscript are included in this article and as part of the SI. The raw data supporting the conclusions of this article will be made available by the authors without any undue reservation. Samples of final compounds are available from the authors upon request.
