# Supplementary figures and images for: Multi-targeted azacoumarin–cyanocinnamate hybrids induce G2/M arrest and apoptosis via tubulin, and COX-2/VEGFR modulation: insights from in vitro mechanistic basis and in vivo validation
Source: RSC Med Chem. 2025 Aug 22;16(11):5574–601. doi: 10.1039/d5md00484e (PMC12461739; doi:10.1039/d5md00484e)

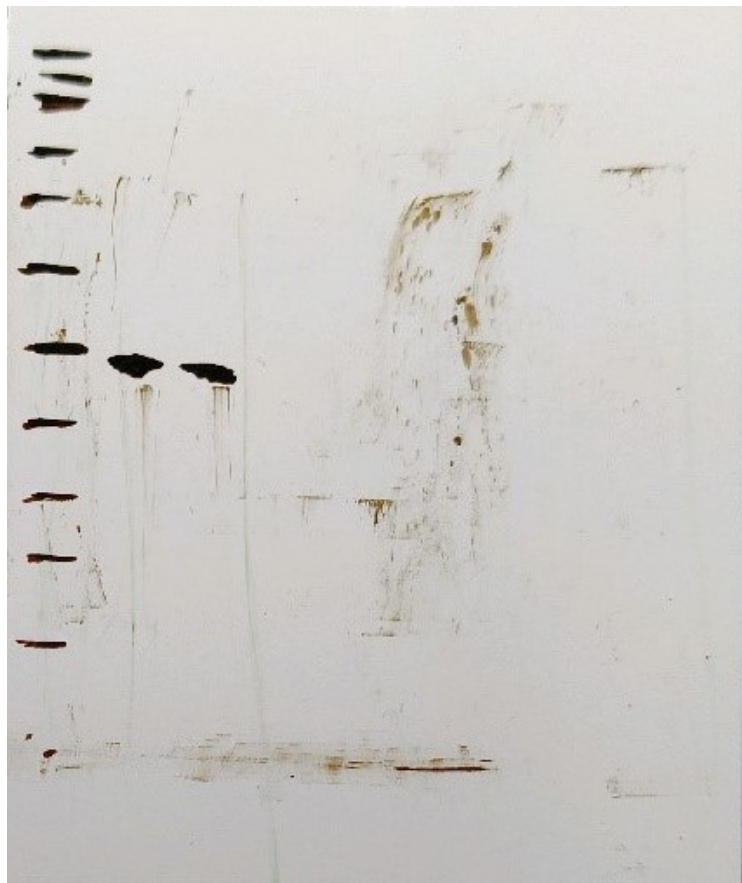

Supplement: MD-016-D5MD00484E-s001 [file MD-016-D5MD00484E-s001.pdf]

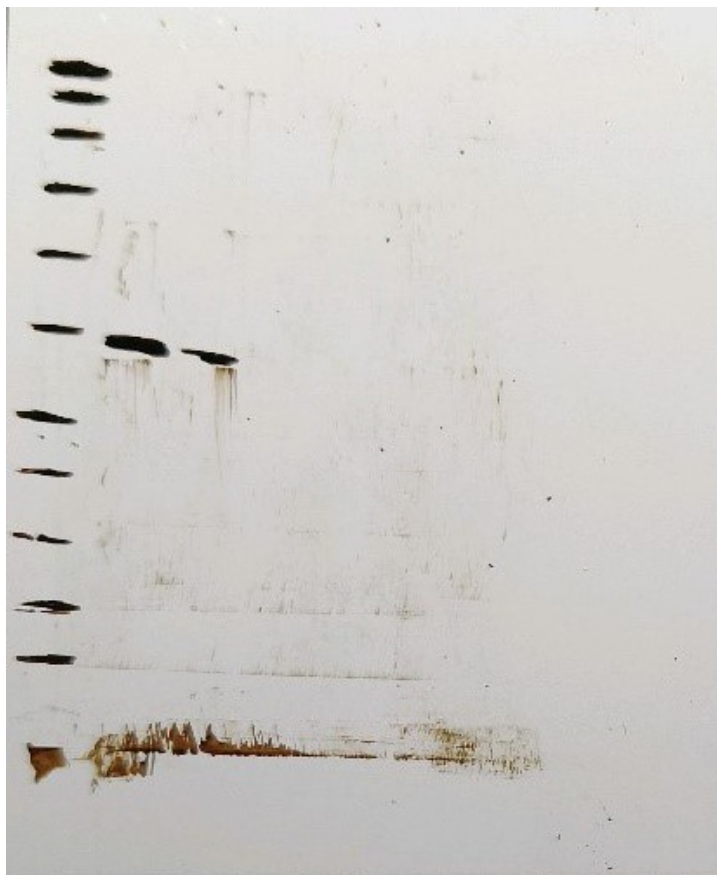

Supplement: MD-016-D5MD00484E-s003 [file MD-016-D5MD00484E-s003.pdf]
