# Supplementary material for: Multi-targeted azacoumarin–cyanocinnamate hybrids induce G2/M arrest and apoptosis via tubulin, and COX-2/VEGFR modulation: insights from in vitro mechanistic basis and in vivo validation
Source: RSC Med Chem. 2025 Aug 22;16(11):5574–601. doi: 10.1039/d5md00484e (PMC12461739; doi:10.1039/d5md00484e)
Supplement: MD-016-D5MD00484E-s002 [file MD-016-D5MD00484E-s002.pdf]

Supplementary Information (SI) for RSC Medicinal Chemistry.
This journal is © The Royal Society of Chemistry 2025

# **Supporting Information**

**Table S1:** Summary of $IC_{50}$ values for the antiproliferative activity of the synthesized compounds (**6a**, **6b**, **7**, **8**, and **9**) and doxorubicin toward MCF-7, MDA-MB 231, and MCF-10A cells.

| **Compd. No.** | **$IC_{50}$ (μM)** | | |
|---|---|---|---|
| | **MCF-7** | **MDA-MB 231** | **MCF-10A** |
| **Compd. 6a** | 26.6±1.56 | 19.6±1.72 | -- |
| **Compd. 6b** | 112.1±6.57 | 31.1±1.87 | -- |
| **Compd. 7** | 7.6±0.45 | 9.7±1.15 | 52.1±1.76 |
| **Compd. 8** | 17.8±1.05 | 26.9±0.79 | -- |
| **Compd. 9** | 33.4±1.96 | 44.2±2.96 | -- |
| **Doxorubicin** | 12.5±0.93 | 8.4±0.61 | 16.2±0.55 |

**Table S2:** The binding score and interactions of compound **7**, native co-crystallized ligands, and positive controls (colchicine, CA-4, celecoxib) toward the tubulin, COX-1, and COX-2 proteins.

| Target | Ligand | Score (kcal/mol) | RMSD (Å) | Key Interacting Residues | Interaction Type(s) |
|---|---|---|---|---|---|
| **Tubulin (PDB:*6pc4*)** | **ABI-274** | -8.92 | 0.12 | Lys252 | Amide-Pi |
| | | | | Leu253 | Pi-sigma |
| | | | | Met257 | Pi-sulfur |
| | **Colchicine** | -6.09 | — | Asn256 | H-bond |
| | **CA-4** | -8.25 | — | Met257 | Pi-sulfur |
| | **Compound 7** | -7.58 | — | Asn256 | H-bond + Amide-Pi |
| | | | | Met257 | Pi-sulfur |
| | | | | Asn348 | Amide-Pi |
| **COX-1 (PDB: *1eqg*)** | **Ibuprofen** | -7.93 | 0.33 | Arg120 | Ionic and salt bridge |
| | | | | Tyr355 | H-bond |
| | **Celecoxib** | -3.97 | — | Arg120 | 2 H-bonds |
| | | | | Tyr355 | H-bond + Pi-sulfur |
| | | | | Gly526 | Amide-Pi |
| | **Compound 7** | -6.28 | — | Arg120 | H-bond |
| | | | | Tyr355 | H-bond |
| | | | | Ser530 | H-bond |
| | | | | Arg83 | Ionic bond |
| **COX-2 (PDB:*4ph9*)** | **Ibuprofen** | -7.47 | 0.14 | Arg121 | H-bond and salt bridge |
| | | | | Tyr356 | H-bond |
| | **Celecoxib** | -8.31 | — | Arg121 | 2 H-bonds + Pi-cation |
| | | | | Tyr356 | 2 H-bonds |
| | | | | Ser354 | H-bond + Pi-sigma |
| | | | | Phe519 | H-bond |
| | | | | Gln193 | H-bond |
| | | | | Ile518 | H-bond |
| | | | | Arg514 | H-bond |
| | | | | Leu353 | H-bond |
| | | | | Gly527 | Amide-Pi |
| | **Compound 7** | -7.09 | — | Arg121 | Salt-bridge + Pi-cation |
| | | | | Tyr356 | Pi-Pi |
| | | | | Lys83 | Pi-cation |
| | | | | Glu525 | Pi-anion |
| | | | | Ser120 | H-bond |

**Table S3:** The list of primers utilized in gene expression assessment using RT-PCR

| Gene | Sequence |
|---|---|
| ***BAX*** | Forward: 5′-GAGGAACTGGACAGTAACATGGAGCT-3′<br>Reverse: 5′-CGGCCCCAGTTGAAGTTGC-3′ |
| ***BCL2*** | Forward: 5′-GCCGGTTCAGGTACTCAGTCATC-3′<br>Reverse: 5′-GTCACCTTCACCGTTCCA-3′ |
| ***TP53*** | Forward: 5′-AGAGTCTATAGGCCCACCCC-3′<br>Reverse: 5′-GCTCGACGCTAGGATCTGAC-3′ |

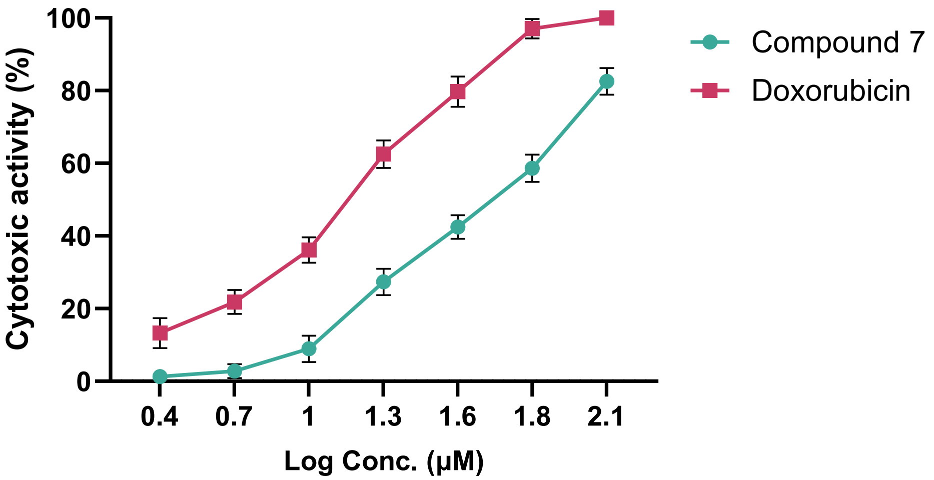


**Figure S1**. *In-vitro* antiproliferative screening of the synthesized compound **7** and doxorubicin toward normal human breast MCF-10A cells. A) Representative dose-response cytotoxic activity of examined compounds. B) Representative $IC_{50}$ values for the antiproliferative activity of examined compounds toward MCF-10a cell viability. Data is presented as mean±SD from, with n=3 experiments.

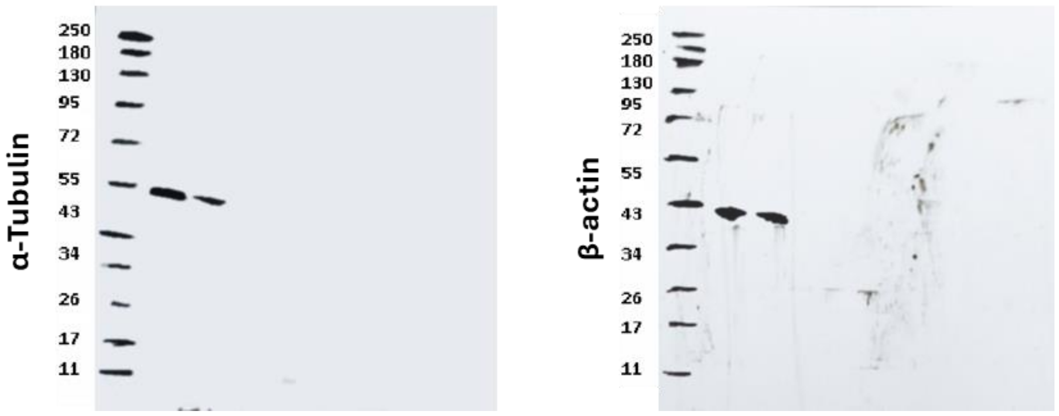


**(A)** **(B)**

**Figure S2**. Effect of compound **7** (at its $IC_{50}$ value) on the expression of tubulin protein in MCF-7 cells.

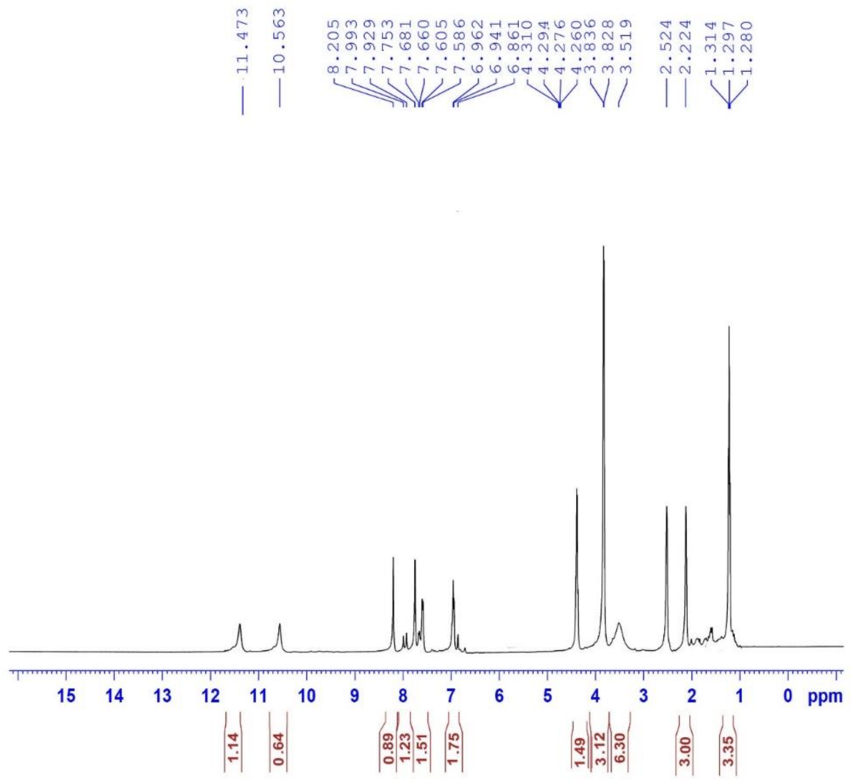

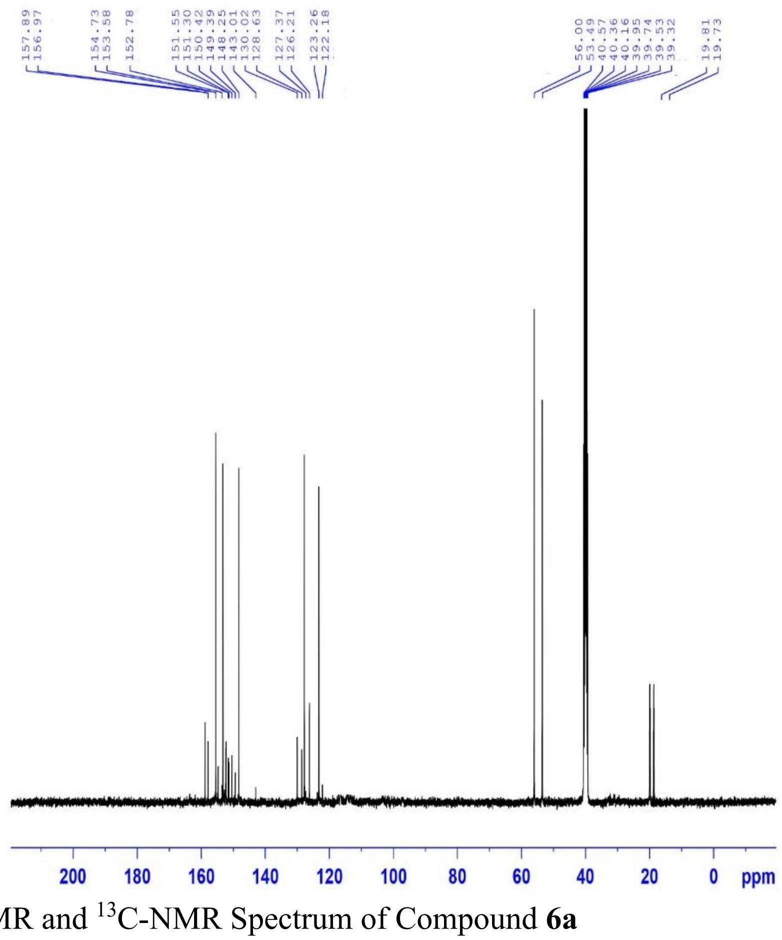

**Figure S3**: $^{1}$H-NMR and $^{13}$C-NMR Spectrum of Compound **6a**

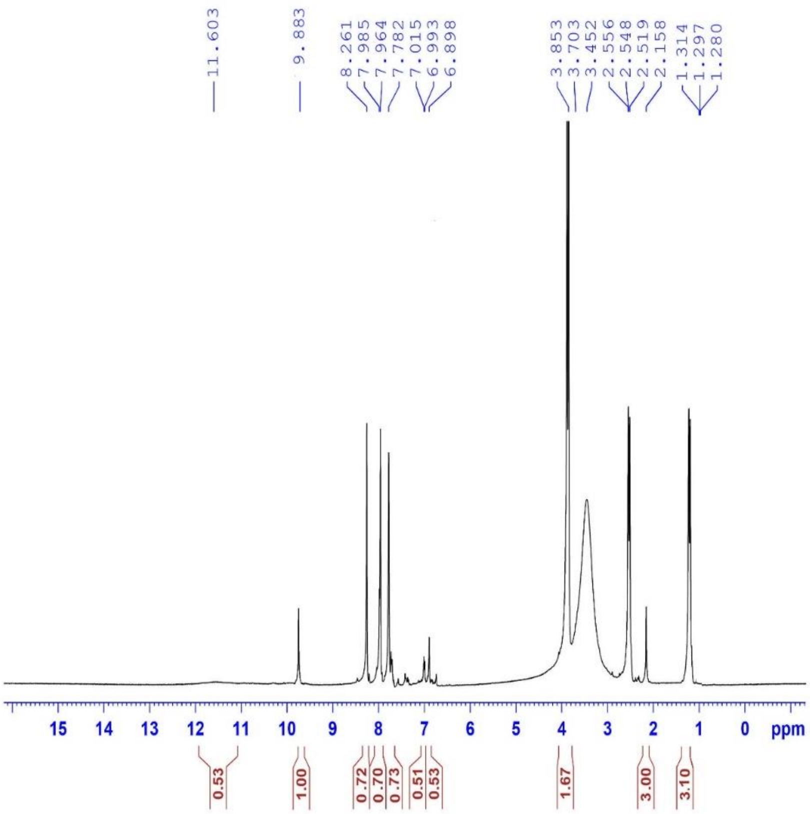


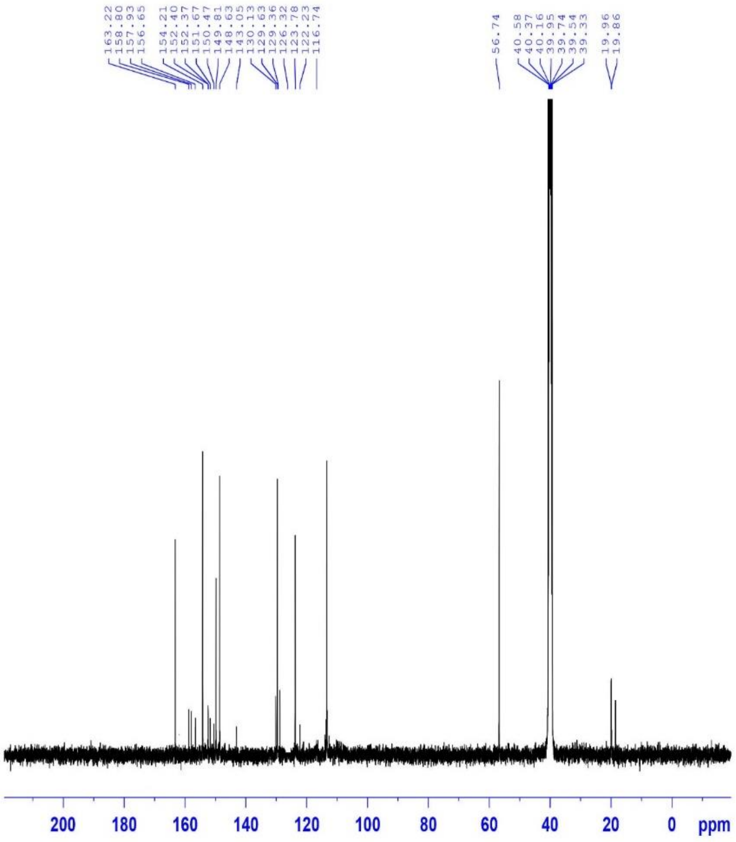


**Figure S4**: $^{1}$H-NMR and $^{13}$C-NMR Spectrum of Compound **6b**

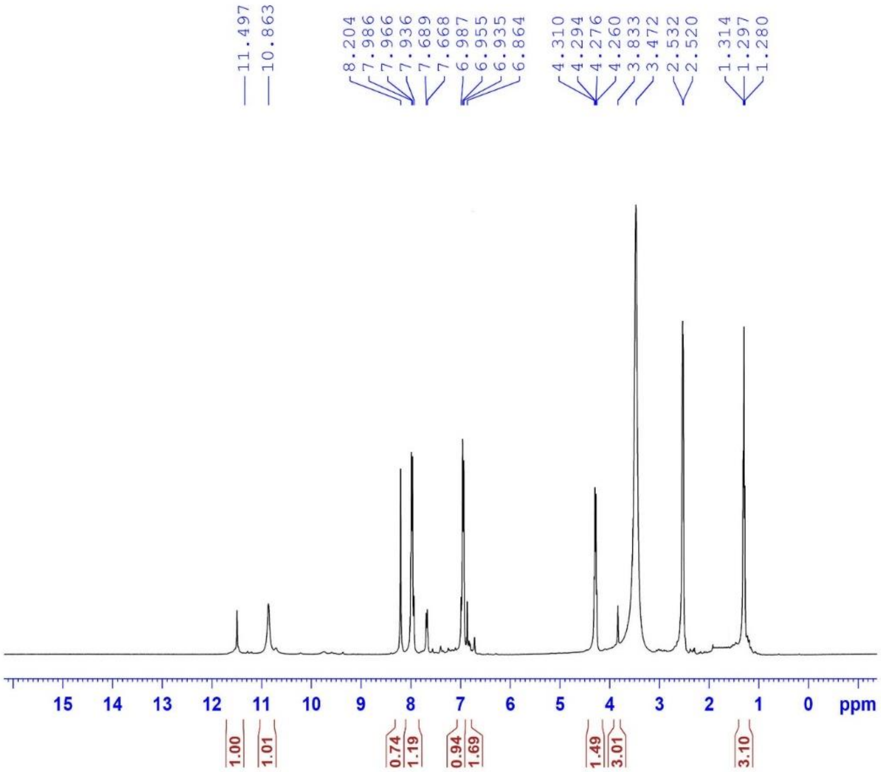


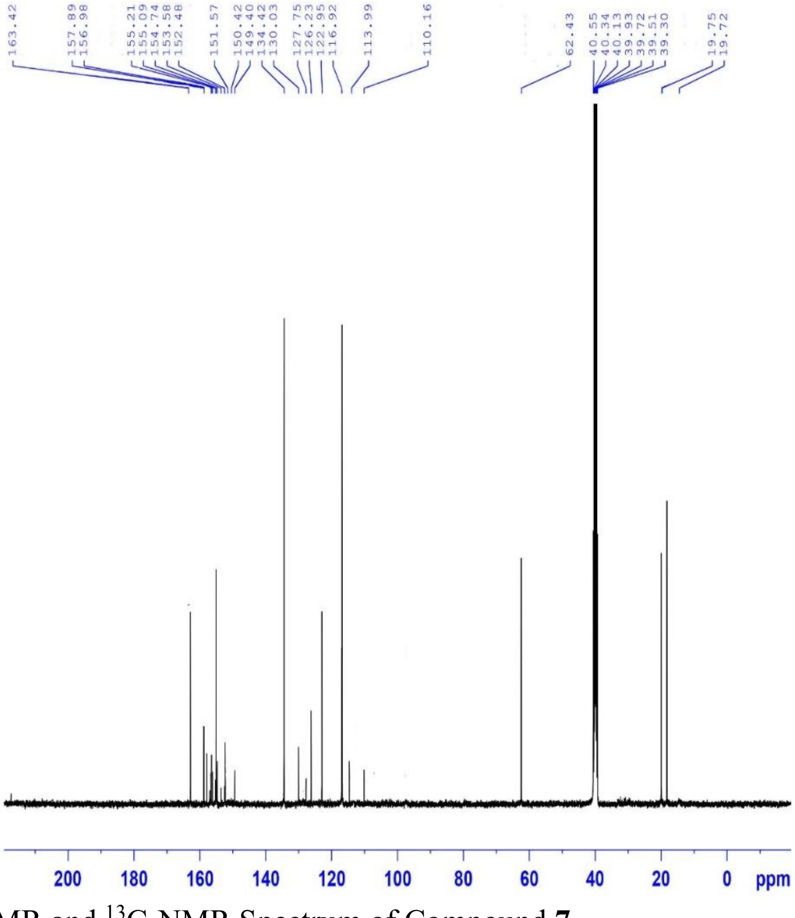


**Figure S5**: $^{1}$H-NMR and $^{13}$C-NMR Spectrum of Compound **7**

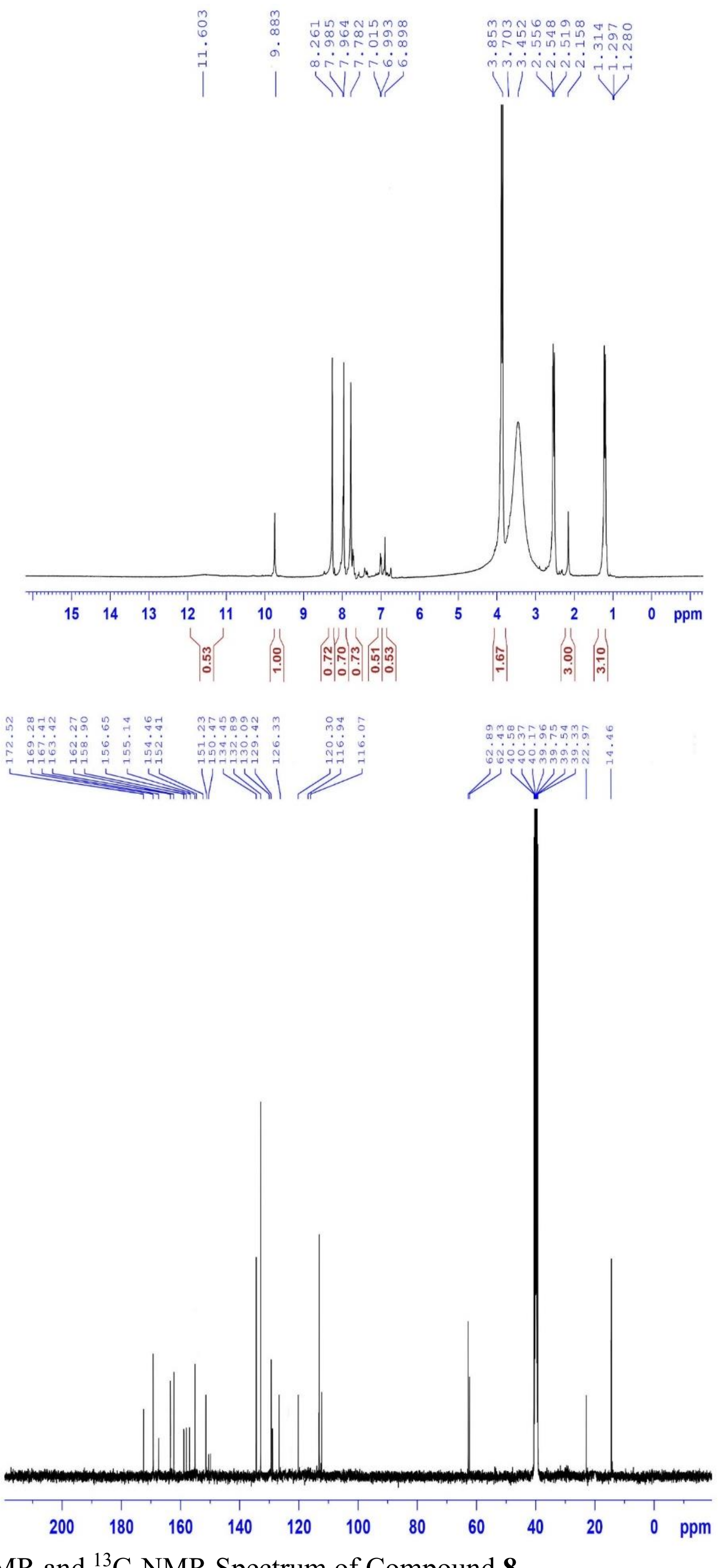

**Figure S6**: $^{1}H$-NMR and $^{13}C$-NMR Spectrum of Compound **8**

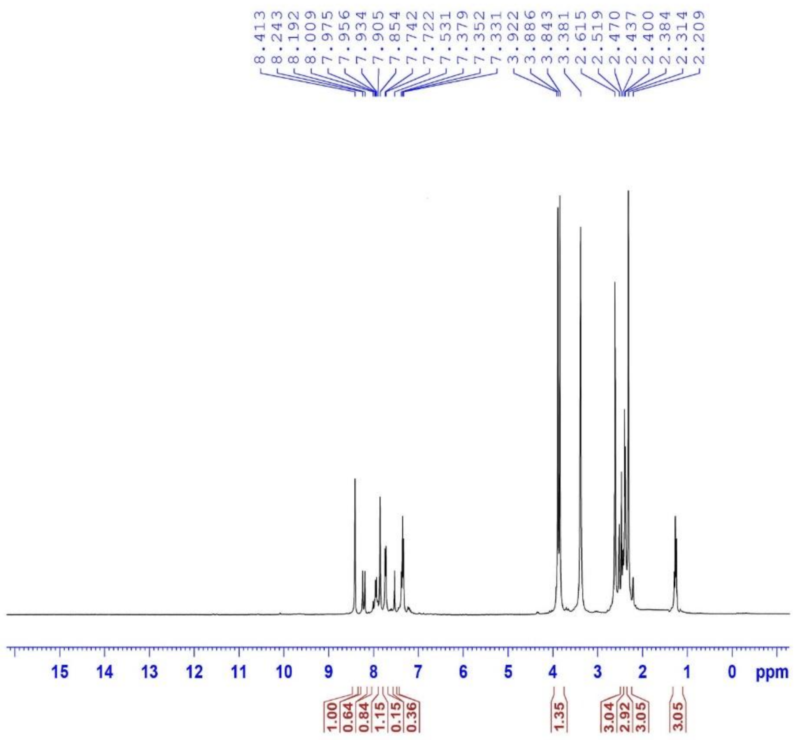

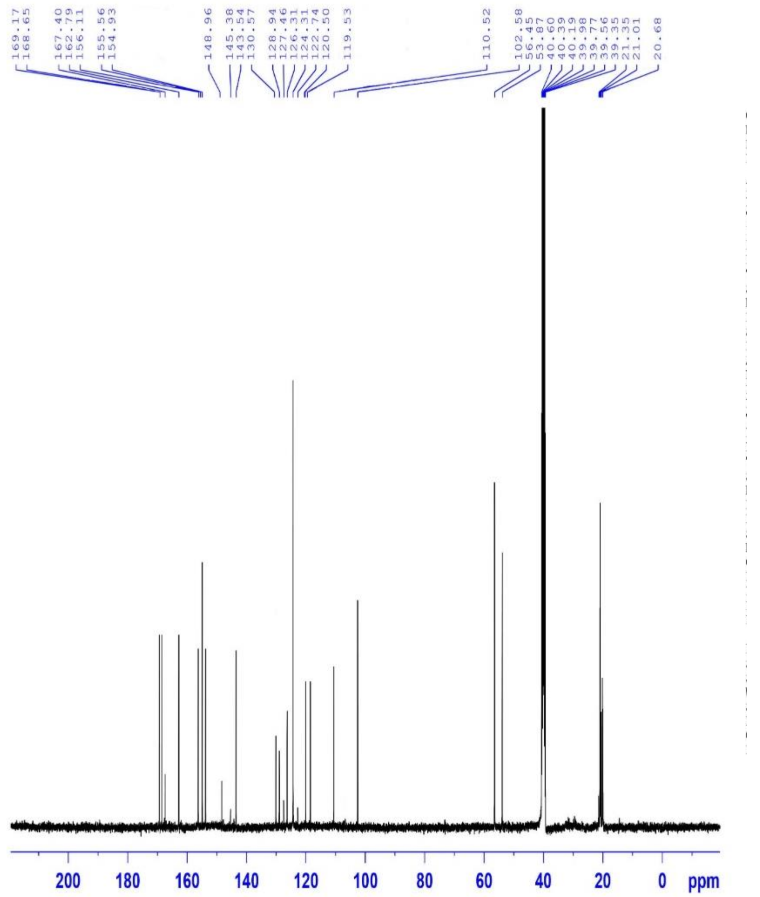

**Figure S7**: $^{1}$H-NMR and $^{13}$C-NMR Spectrum of Compound **9**
